# Phenotypic Profiling of SOD1-G93A Mice: Integrating Sciatic Nerve Histopathology with Systemic Chemokine Dynamics During ALS Progression

**DOI:** 10.3390/ijms27198604

**Published:** 2026-09-25

**Authors:** Ekaterina Martynova, Marat Mukhamedyarov, Emmanuel Kabwe, Svatlana Khaiboullina, Owen Simwalangana, Maria Markelova, Albert Rizvanov, Svetlana Arkhipova, Ilnur Salafutdinov

**Affiliations:** 1 Institute of Fundamental Medicine and Biology, Kazan Federal University, Kazan 420008, Russia; marat.muhamedyarov@kazangmu.ru (M.M.); emmanuelkabwe@ymail.com (E.K.); rizvanov@gmail.com (A.R.);; 2 Department of Normal Physiology, Institute of Neurosciences, Kazan State Medical University, Kazan 420012, Russia; 3 Department of Medical Biology and Genetics, Kazan State Medical University, Kazan 420012, Russia; 4 Department of Chemistry and Biology, School of Natural and Applied Sciences, Mulungushi University, Kabwe P.O. Box 80415, Zambia

**Keywords:** neurodegeneration, amyotrophic lateral sclerosis, SOD1-G93A mice, astrocytes, microglial cells, demyelination, cytokine multiplex analysis, myelin fibers, ultrastructural analysis, peripheral nerve, spinal cord

## Abstract

Amyotrophic lateral sclerosis (ALS) is a devastating neurodegenerative disorder characterized by the progressive loss of motor neurons, leading to muscle atrophy and fatal respiratory failure. While neuroinflammation is a hallmark of ALS, the temporal and spatial relationship between systemic inflammation and peripheral nerve degeneration remains poorly understood. In the present study, the transgenic SOD1-G93A mouse model was employed to conduct a longitudinal analysis of amyotrophic lateral sclerosis (ALS) progression. We integrated morphometric and ultrastructural examinations of the sciatic nerve with immunofluorescent profiling of the spinal cord (GFAP, IBA1, NG2). In addition, cytokine and chemokine dynamics were analyzed using 33-plex multiplex assay in the serum at various stages of the disease. Histological analysis revealed a progressive decline in myelin fiber density within the sciatic nerve and axonal death. As the disease progressed, Schwann cells exhibited morphological features consistent with a pro-fibrotic shift, as suggested by collagen accumulation observed on TEM. However, direct confirmation of a fibrotic phenotype requires additional markers (e.g., Col I/III, α-SMA, TGF-β) and was not assessed in this study. Significant motor neuron loss and robust glial activation were demonstrated in the spinal cord. Blood plasma analysis identified a coordinated systemic up-regulation of MCP-3, Eotaxin, CTACK, and TECK. There was lack of inflammatory cell infiltration in the peripheral nerve tissues. Our results indicate that ALS-associated peripheral nerve degeneration in the SOD1-G93A mice model is primarily a degenerative process characterized by early demyelination and without leukocyte infiltration. However, increased levels of MCP-3, Eotaxin, CTACK, and TECK are associated with disease progression and may reflect ongoing central neuroinflammation. While these changes are temporally consistent with blood–spinal cord barrier dysfunction reported in SOD1 mice, direct assessment of barrier integrity was not performed in this study. These data highlight the potential of blood chemokines as step biomarkers and highlight the compartmental nature of inflammation in ALS, where systemic immune changes do not necessarily equate to local inflammation in the peripheral nerve.

## 1. Introduction

ALS is an idiopathic neurodegenerative disease accompanied by the progressive death of both upper motor neurons (UMN) in the brain and lower motor neurons (LMN) in the spinal cord/brainstem, leading to the loss of motor function, disability, and death within 5 years [1]. Currently, there is no curative treatment for ALS; however, several therapeutic strategies, such as riluzole and edaravone, have been approved and are used in clinical practice to slow disease progression and alleviate its clinical course, though their efficacy remains limited [2]. The lack of precise diagnostic tests and specific biomarkers that allow for unequivocal early identification of the disease further complicates clinical management and highlights the urgent need for a deeper understanding of ALS pathogenesis [3]. A more detailed exploration of the molecular mechanisms underlying ALS reveals a complex interplay of genetic and cellular factors. In this context, glutamate excitotoxicity, oxidative stress, impaired axonal transport, and increased intracellular Ca2+ levels are well-established mechanisms contributing to neurodegeneration [4]. Furthermore, mutations in several genes have been strongly linked to both familial and sporadic forms of ALS, including SOD1, TARDBP, FUS, ANG, and OPTN, underscoring the genetic heterogeneity of the disease and pointing to diverse pathogenic pathways such as protein misfolding, RNA metabolism disruption, and impaired autophagic clearance. Data also suggest that mitochondrial dysfunction also plays an important role in motor neuron degeneration [5]. Ultrastructural analysis of skeletal muscle cell mitochondria demonstrated fragmentation, enlargement of cristae, changes in the shape of mitochondria, and paracrystalline inclusions in hepatocytes of ALS [6].

Dysregulation of immune mechanisms was demonstrated in ALS. Cytokine activation was suggested to regulate the neuroinflammation in ALS. Changes in pro-inflammatory and anti-inflammatory cytokines as well as growth factors were reported in ALS [7]. Also, a marked activation of microglia and astrocytes producing neuroprotective and pro-inflammatory molecules capable of affecting the rate of motor neuron degeneration was shown in ALS [8]. Brain infiltration with CD4+ and CD8+ T-lymphocytes was shown in ALS and animal models [9]. However, the data remain fragmented making the comprehensive understanding of ALS pathogenesis difficult.

This study aimed to establish a comprehensive understanding of the ALS pathogenesis using the SOD1-G93A mice model. We investigated the relationship between central nervous system neuroinflammation, and cell populations (astrocytes, microglial cells, NG2 cells, fibroblasts) infiltrating the spinal cord. Also, the correlation between infiltrating cell type, degeneration of peripheral nerves, and circulating cytokine dynamics was analyzed in all stages of mice ALS model.

## 2. Results

### 2.1. Light Microscopy Assessment of Myelin Fiber Count Dynamics in the Sciatic Nerve

The histological and the anatomical structure of the sciatic nerve present major changes as ALS progresses. In wild-type mice, the sciatic nerve consists of two large bundles of myelin fibers surrounded by perineurium, also named a first-order bundle. Sometimes, there was an additional beam of the first order fibers found with smaller diameter. The average number of myelin fibers in the sciatic nerve in wild type mice was 5890 ± 433.

At the stage of preclinical manifestations, the sciatic nerve also had two large and one small bundle of first order fibers surrounded by epineurium. However, a small number of myelin fibers appeared, having a hypertrophic structure with signs of demyelination. Also, a decreased number of myelin fibers by 61.2% (2285.66 ± 240.6) as compared to wild-type mice (5890 ± 433) was found (*p* < 0.05).

At the stage of clinical manifestations, the peripheral nerve was divided into 7–8 bundles of the first order with a much smaller diameter. Some myelin fibers became hypertrophic and stained intensely with methylene blue, while others remained similar to those in the control group (Figure 1A). The number of intact myelin fibers at the clinical stage was significantly lower (1095.33 ±218.6; 52.1%) than at the preclinical stage (2285.66 ± 240.6) and in wild-type (5890 ± 433) mice (81.4%).

In mice with the terminal stage, the sciatic nerve remained filamentous and separated into 5–7 bundles of the first order with smaller diameter. Almost all myelin fibers appeared damaged, widely spread with overgrown endoneurium between bundles. The number of myelinated fibers significantly decreased compared to all other studied groups and amounted to 8.6% (1012.66 ± 150.3; *p* < 0.05).

Myelin fiber counts were decreased compared to all other groups at 8.6% (1012.66 ± 150.3; *p* < 0.05). We concluded that disease progression is characterized by degeneration and a gradual decrease of myelin fiber counts in the sciatic nerve in mice with ALS.

### 2.2. Transmission Electron Microscopy

Figure 2 shows microphotographs of the intact sciatic nerve of a wild-type mouse: a general view (Figure 2), in which myelinated fibers (Figure 2), unmyelinated fibers, and Schwann cells (Figure 2A’ magnified area) can be distinguished. This served as a control for all other groups. The ultrastructural organization of the peripheral nerve in all other groups was described in comparison with this control. In the sciatic nerve, myelin fibers were closely located at the stage of preclinical manifestation. Individual cells, fibroblasts and microglia, were rarely found between myelin fibers and clusters of degenerating tissue (Figure 3A). Fibroblast growth between myelin fibers was noted. Connective tissue was found to grow, especially in areas where degenerating elements were present (Figure 3B).

Among the myelinated fibers, the perikarya of Schwann cells can be identified; some of them form myelinated, others form unmyelinated fibers (arrow) (Figure 3A,D).

Schwann cell perikarya are observed among nerve fibers; certain cells generate myelinated fibers, whereas others generate unmyelinated fibers. Axons maintained structural integrity. Mainly mitochondria and microtubules were found in the axon cytoplasm (Figure 3C). Also, myelin layers remained partially visible with electron-dense formations between the layers. These formations could be products of myelin breakdown. They often crumble out during sample preparation (myelin ovoids) (Figure 3A’, red arrow). The basement membranes surrounding the Schwann cells remain intact and clearly visible (Figure 3C, blue arrow). The cytoplasm and nuclei of Schwann cells also remained unaffected similar to wild controls (Figure 3A,A’). However, the individual myelin fibers had changed morphology: fibers had signs of demyelination as follows: (1) 3 times thicker myelin layer; (2) compression of the axons with significant size reduction compared to wild control; (3) presence of cavities in the cytoplasm indicating axon degeneration. Also, a large number of myelin bundles appear in the myelin sheath, so the myelin index was 4/1 (Figure 3D).

At the stage of clinical manifestations, electron microscopy revealed substantial separation of the myelin sheaths compared to the preclinical stage. In addition, large electron-dense conglomerates consisting of degenerated nerve elements (myelin ovoids) were present (Figure 4A,B). However, the cytoplasm of myelin-forming Schwann cells showed signs of degeneration: vacuolation, absence of organelles, and filamentous basement membrane (arrow) (Figure 4C). The axon also had signs of microtubule disintegration. Layers of connective tissue between myelin fibers became more prominent with large bundles of collagen fibers (arrow) becoming visual (Figure 4B,D).

Sciatic nerve, terminal stage of disease. In fascicles with residual fibers, preserved axons are sparsely and unevenly distributed within large intervals filled with connective tissue (Figure 5A–C). Myelin sheaths are uniformly electron-lucent; individual myelin lamellae are not discernible. Numerous large electron-dense aggregates are present within myelin (Figure 5D). In the connective tissue between degenerating myelinated fibers, numerous cells with oval nuclei, euchromatin, and prominent nucleoli—consistent with fibroblasts—are evident, indicating active fibrosis with replacement of neural tissue by connective tissue (Figure 5A’,D). The question of peripheral nerve fibrosis remains open and requires additional research, which we plan to conduct in the future on this model using various more evidence-based methods. Schwann cell cytoplasm lacks visible organelles; basal laminae are disrupted and fragmented (Figure 5E).

Spinal cord. In mice with preclinical stage of disease the area of preserved spinal cord tissue decreased by 38%, while the area of degenerated gray matter increased (19 µm^2^) compared to wild-type mice spinal cord (Figure 6A,B). In the clinical phase, the area of undamaged spinal cord tissue was significantly reduced by 61%, while the area of degraded material was significantly increased by 12 times, reaching 229 µm^2^ compared to the preclinical phase (19 µm^2^). At the ALS stage, the area of unaffected spinal cord tissue decreased significantly by 39%, while the area of degraded material increased significantly by 23%, reaching 283 µm^2^ compared to the preclinical stage (19 µm^2^).

Spinal cord. Transmission electron microscopy revealed no signs of degeneration in the anterior horn gray matter in wild-type mic (highlighted in red Figure 7A). Motor neurons (MNs) had no signs of degeneration and contained a rounded nucleus with a clear karyolemma (arrow) (Figure 7A). In the cytoplasm of MNs, rough ER cisterns and the Golgi complex were well developed. A large number of rounded mitochondria with clearly developed cristae were found (Figure 7B). MNs were located in gray matter surrounded by unmyelinated and myelinated fibers (Figure 7D) (enlarged area shows the structure of myelinated fibers with clear myelinated layers). In the anterior horn gray matter of the spinal cord, a few non-activated microglial cells were also found: an irregular oval shape, without processes, about 2 µm in diameter, with an oval-shaped nucleus containing heterochromatin, and a tortuous karyolemma (Figure 7C). Inactive astrocytes were also found in the gray matter of the anterior horns. These cells had an irregular polygonal shape, with a cytoplasm of medium electron density, 4 µm in diameter, and an irregular shape nucleus containing heterochromatin (Figure 7D). In the gray matter of the spinal cord of wild-type mice, a large number of oligodendrocytes were found. These were myelin-forming cells with a strictly oval nucleus and electron-bright cytoplasm (Figure 7D).

Ultrastructural analysis of the anterior horn gray matter of the spinal cord of mice with the ALS phenotype revealed impaired tissue ultrastructural integrity (Figure 8A). Specifically, we found partially disintegrated (marked with an arrow) or completely degraded (*) nerve fibers (enlarged area). Also, MNs had signs of apoptosis. The nuclei of apoptotic cells displayed a characteristic irregular (scalloped) nuclear border and granular chromatin pattern and cytoplasmic vacuoles. Additionally, we found reactive astrocytes (RA). RA was characterized by an irregular pyramidal form, electron-dense cytoplasm, and prominent development of the endoplasmic reticulum. (Figure 8B). RA were abundant with an irregular pyramidal shape irregular pyramidal form (Figure 8B), an electron-dense cytoplasm, a well-developed endoplasmic reticulum (ER) (marked by an arrow), and of 18 µm diameter. It could be suggested that, at this stage of the disease, astrocytes become activated similar to that found in brain injury cases.

Ultrastructural analysis of the anterior horn gray matter at the symptomatic stage showed presence of a small number of degenerated MNs with disintegrated cytoplasm and completely degenerated nerve fibers (enlightened areas (*)) (Figure 9A). RA were less numerous and had an irregular shape with medium electron density cytoplasm (Figure 9B). Microglial cell extensions enveloped electron-lucent areas and established direct contact with partially degraded fibers (indicated by arrow). The extensions of microglial cells surrounded the electron-lucent areas and had close contact with partially disintegrated fibers (marked with an arrow). Additionally, degeneration of oligodendrocytes (Ol) was found as characterized by clear cytoplasm and adjacent myelin fibers (enlarged area) with signs of degeneration (myelin layers are not identified) (Figure 9C).

Ultrastructural analysis of the anterior horn gray matter of the spinal cord at the terminal stage of the disease showed significant degeneration in the nervous tissue structure (Figure 10A). The gray matter consisted of a disintegrated matrix (marked with an arrow) that looks homogeneous. The outlines of processes were absent. Activated microglial cells, inactive astrocytes (Figure 10B), and isolated demyelinated fibers (hypertrophy type) were found among extensive lucid areas without any structures. Cells of unknown origin were also identified. These cells may be implicated in neurodegeneration, given their electron-dense heterochromatic nucleus. (NG2-glial cells) (Figure 10C).

### 2.3. IF Study

#### 2.3.1. GFAP and Iba1 IF

In the anterior horn of the gray matter of the spinal cord in wild-type mice (Figure 11a), grey matter structures were preserved and had no lesions, scars, and demyelinated areas. GFAP+ cells had a compact morphology and were evenly distributed in small numbers within the tissue. Single Iba+ cells were also found, having small sizes and few branching processes. In the anterior horn of the gray matter of the spinal cord in SOD1-G93A mice at the preclinical stage of ALS disease, more GFAP+ cells and Iba1+ cells were found (*p* < 0.05) (Figure 11f), while the bodies of RA and reactive microglia had signs of hypertrophy with more branched processes (Figure 2B).

At the clinical stage of the disease RA counts increased significantly (*p* < 0.05) compared to the preclinical stage. Cells were located individually and in clusters around dying MNs and their processes (Figure 11). The hypertrophy of RA was enhanced, and they also were processed. The number of Iba1+ microglial cells (5 ± 2) remained unchanged compared with the preclinical stage (5 ± 2) and control (5 ± 2) (Figure 11f). In addition, the shape of these cells remained similar to that in the preclinical stage (Figure 11c).

At the terminal stage of the disease, damage to the structures of the gray matter of the spinal cord was most prominently pronounced, and visually visible areas of demyelination and death of motor neurons were significantly numerous. Expression of Iba1 was detected in numerous cells with simplified morphology: the cells had an oval perikaryon and 2–3 processes. GFAP+ astrocytes cells were located both at the edges of the damaged areas and degenerated neurons and as individual cells, with numerous processes penetrating the surrounding tissues (Figure 11d,e).

#### 2.3.2. OLIG2 and NG2 IF

A few NG2+ cells were found in the spinal cord tissue of control mice (Figure 12e). Oligodendrocytes were also located in the spinal cord of the control spinal cord. These cells expressed transcription factor (Olig2) in the nucleus (Figure 12f).

NG2+ cells and oligodendrocytes were also found in the spinal cord tissue of mice in the preclinical stage of ALS (Figure 12d–f). At the clinical stage, Olig2 expression cell amount was reduced in the spinal cord нa 78.6%. Also, the NG2 proteoglycan (antibodies Olig-2 anti-Mouse 1:200, Santa Cruz, Dallas, TX, USA) was found in the cytoplasm of Olig2+ cells (Figure 12d–f). Additionally, the expression of NG2 proteoglycan was enhanced in the cytoplasm of Olig2+ cells (Figure 12d–f).

### 2.4. Dynamics of Serum Cytokine Levels in Mice with ALS

We analyzed 33 cytokines, including pro- and anti-inflammatory cytokines, chemokines, and growth factors, in the blood plasma of transgenic SOD1-G93A mice at various stages of the disease. A comprehensive list of all 33 analyzed cytokines, along with their quantitative values at each disease stage, is presented in Supplementary Table S1. Our analysis revealed a distinct systemic inflammatory signature characterized by up-regulation of specific chemokines. MCP-3 (CCL7) was increased in asymptomatic and symptomatic stages, suggesting early recruitment of monocytes. Elevated Eotaxin (CCL11) was detected throughout the disease course. Levels of CTACK (CCL27) and TECK (CCL25) were elevated during the terminal stage of the disease, suggesting a profound systemic immune activation (Figure 13).

It should be noted that while changes in cytokine levels were prominent in the blood plasma, there was limited cell infiltration in the sciatic nerve of SOD1-G93A mice (Figure 13). These data suggest a presence of compartmentalized immune response, where systemic chemokine signaling may reflect central nervous system (CNS) pathology or generalized distress, rather than localized inflammation within the peripheral nerves themselves.

## 3. Discussion

### 3.1. Distal Axonopathy and Ultrastructural Changes in the Peripheral Nerve

One hypothesis explaining pathogenesis of ALS states is that it is a distal axonopathy [10]. According to this hypothesis, the death of motor neurons in the spinal cord begins with their axons. Thus, the absence of neuronal death or morphological changes in motor neuron cell bodies was described later in life, in 60-day-old presymptomatic SOD1 mice [10,11,12]. However, histopathological examination of the sciatic nerve in 60-day-old presymptomatic SOD1 mice revealed no difference in structure from the sciatic nerve of wild-type mice using light microscopy [13]. Therefore, we used electron microscopy to analyze the ultrastructural changes in the sciatic nerve of ALS mice. We found that, in the presymptomatic stage, ALS mice have ultrastructural signs of myelin degeneration, such as myelin ovoids and partial disintegration of microtubules in axial cylinders. In contrast, the cytoplasm of Schwann cells displayed limited changes.

### 3.2. Glial Activation Dynamics in the Spinal Cord

The increase in the number of astrocytic glia in pre-symptomatic 60-day-old mice in our study correlates in timing with an early increase in the cytokine MCP-3, which is associated with the activation of astrocytic glia in some brain pathologies, in particular encephalitis [14]. Reactive astrocytes attract microglia, and at subsequent stages, we observed an increase in its levels, specifically phagocytic microglia, for which Iba1 is a marker. Thus, the lack of dynamics of Iba1+ microglial cells in the anterior horn of the gray matter of the spinal cord in the preclinical and clinical experimental groups was demonstrated in our study, and their significant increase at the terminal stage is most likely due to the fact that, at the terminal stage, microglial cells are activated under the influence of astrocytic signals and transform into a phagocytic phenotype. This coincides with a decrease in the number of astrocyte glia, which phagocytic microglial cells destroy, as well as destroying the remaining cellular debris and degenerated tissue of the spinal cord. Thus, the MCP-3 molecule may be a potential target for reducing neuroinflammation and gliosis that accompany this disease. The microglial response observed in our study is consistent with the concept of two-phase microglial activation in ALS, in which an early neuroprotective phase is followed by a later neurotoxic phase [15,16]. The wave-like dynamics of astrocyte activity—with a peak at the clinical stage and a decline at the terminal stage—may reflect the transition from a neuroprotective to a neurotoxic astrocyte phenotype, as previously shown in the work by Liddelow et al. [17]. The interaction between astrocyte and microglia activation requires further study using stage-specific markers of glial polarization.

The results of our study demonstrate a clear temporal correlation between astrocyte activation, reflected in the increased number of GFAP+ cells, and the elevation of MCP-3 cytokine levels, which suggests a potential key role of this cytokine in the early neuroinflammatory response in ALS pathogenesis, as previously reported in neuroinflammatory conditions [14,18]. However, further research is required to fully elucidate the causal relationship between these observations and to determine whether MCP-3 acts as a primary driver or a secondary mediator in this process.

The early stages of ALS pathogeneses changes are believed to be primarily in axon transport, which present with the disintegration of the axon cytoskeleton, its fragmentation, and decreased mitochondria function. These are events commonly reported during Wallerian degeneration of the peripheral nerve. Schwann cells become engaged later, once they become denervated. Some of these cells transform to phagocytes others undergo a pro-fibrotic shift, as suggested by collagen accumulation observed on TEM; however, direct confirmation of a fibrotic phenotype requires additional markers Col I/III, α-SMA, TGF-β and was not assessed in this study.

In our study, Schwan cells appear to have a phagocytic function in both stages, clinical manifestations, and the terminal stage. The role of these altered Schwann cells in ALS pathogenesis is unclear; they may recruit macrophages, since recent studies have shown that one hundred Schwann cells can initiate peripheral nerve inflammation via expression of CSF1, IL-34, and SCF [19,20]. Increased expression of K67 on Schwann cells at the stage of clinical manifestations was demonstrated. These could be Schwann cells dividing and forming Bünger’s ribbons, as in Wallerian’ s degeneration. It should be noted that sensory nerves were shown to be damaged in ALS as well as in transgenic mice [19]. However, damage to sensory nerves was less pronounced compared to motor nerves. This may explain the presence of some of the surviving axons at the terminal stage in our study.

### 3.3. Systemic Cytokine and Chemokine Response

Meta-analysis of 66 studies using the SOD1-G93A mouse model demonstrated increased multiple cytokines and a progressive imbalance between pro- and anti-inflammatory responses [21] However, some studies report an absence of changes in cytokine levels in ALS [22,23,24]. In our study, an increased level of four chemokines was found in SOD1-G93A mice as the disease progressed. Of note, increased levels of CTACK, MIP-3α, and TECK were found. Our finding of early MCP-3 elevation aligns with the early glial activation reported by Henkel et al. (2004) [18]. In contrast, the terminal surge of TECK and CTACK is temporally associated with the blood–spinal cord barrier dysfunction described by Zhong et al. [25], who demonstrated that vascular changes and barrier breakdown occur prior to motor neuron loss in SOD1 mice. However, as BSCB integrity was not directly assessed in our study, this association remains speculative. Notably, while Zhong et al. [25] found that MCP-1 (CCL2) elevation follows barrier disruption, our data show that MCP-3 is elevated earlier, suggesting distinct temporal roles for different chemokines in ALS pathogenesis.

Our data on changes in Eotaxin level correlate with the results of Moreno-Martínez et al. (2019) [23], who also reported elevation of these cytokines at the asymptomatic stage. Increased Eotaxin has been linked to microglial activation and proliferation in neurodegenerative diseases [26,27]. In our study, the number of microglial cells in the spinal cord was also increased. This supports the assumption that immune reactions are involved in the pathogenesis of ALS before overt symptoms appear.

The light and electron microscopy methods used did not reveal any morphological signs of inflammation in the examined sections of the sciatic nerves. Given that transmission electron microscopy covers very small areas of the study, this does not allow for definitive conclusions about the absence of neuroinflammation. This data is consistent with the concept of a ‘dying-back’ axonopathy with demyelination and a pro-fibrotic shift [19,28]. However, it is important to note that studies by Angelini et al. [20] demonstrated a very early involvement of innate immunity in the peripheral nerve’s pathology in SOD1-G93A mice. This involvement was characterized by the presence of mast cells and macrophages, as well as IgG deposition, which precedes and may contribute to Wallerian-like degeneration [20]. Thus, while the neurodegenerative process in sciatic nerves may progress without massive, sustained inflammation, our data, in line with Angelini et al. [20], suggest that initial, localized immune events within the nerve itself could play a crucial role in triggering the axonopathy characteristic of ALS.

## 4. Methods

### 4.1. Animals and Ethical Approval

Fifteen 6-week-old ALS phenotype expressing SOD1 transgenic (B6SJL-TG(SOD1-G93 A)dl1Gur/J) mice [29,30] and five C57BL/6J wild type mice (15–20 g) were used. Animals of both sexes were included in all groups, randomly. Thus, animals of both sexes were assigned to each experimental group. All experiments were carried out in compliance with the order and requirements approved by the Ministry of Health of the Russian Federation (On order of the Ministry of Health of the Russian Federation of 23 August 2010 № 708н); and procedural protocols approved by the Kazan Federal University (KFU) local ethics committee (protocol #5, date: 27 May 2014) according to the rules adopted by KFU and Russian Federation Laws and Directive 2010/63/EU of the European Parliament and of the Council of 22 September 2010.

Mice were maintained in separate shoebox-type cages with food and water ad libitum. The room was maintained on a light–dark cycle with standard room temperature and humidity.

Mice were divided into groups: 1—30 day-old SOD1-G93A transgenic mice, the preclinical stage of ALS (no clinical signs, neurological score 4: full extension of hind limbs when suspended by the tail, no signs of motor dysfunction; body weight comparable to wild-type littermates); 2—60 day-old SOD1-G93A transgenic mice; the clinical stage of ALS (visible motor deficit, neurological score 3: trembling of hind limbs during tail suspension and/or partial collapse of leg extension, visible motor deficit, early weight loss); 3—90 day-old SOD1-G93A transgenic mice; the terminal stage of ALS (weight-loss, paraplegia, and tetraplegia, neurological score 0–1: inability to right within 30 s after being placed on the side, weight loss, paraplegia, and tetraplegia); 4—wild-type control animals (Figure 14). Neurological scoring was performed according to the scale developed by Weydt et al. [31]. Behavioral and functional tests on transgenic mice were conducted and published by us earlier [30].

Blood was collected from the tail vein at each stage of disease progression. Animals were euthanized by decapitation following intraperitoneal injection of a 6.4% chloral hydrate (C_2_H_3_Cl_3_O_2_) solution (Sigma-Aldrich, St. Louis, MO, USA) at a dose of 300 mg/kg, in accordance with procedures approved by the KFU Local Ethics Committee (protocol No. 5, 27 May 2014). Under isoflurane anesthesia, a 2-mm segment was excised at the mid-thigh level (15–20 mm distal to the sciatic notch) via a posterior approach. The L3–L5 segments (lumbar enlargement) were harvested en bloc by dorsal laminectomy and gentle caudal extrusion to avoid compression damage. The tissue was immediately immersed in 2.5% glutaraldehyde in 0.1 M phosphate buffer (pH 7.4) to ensure rapid stabilization of the ultrastructure.

### 4.2. Transmission Electron Microscopy

All samples (spinal cord and sciatic nerve) were fixed in 2.5% glutaraldehyde in 100 мM PBS (pH 7.3) for 24 h at 4 °C. Post-fixation, tissue was treated with 1% osmium tetroxide for 2 h and dehydrated using series of ethanol at 30 to 96% *v*/*v* concentrations for 10 min, acetone, and the final step including propylene oxide for 30 min before embedding in Epon 812 resin. After resin polymerization at 37 °C, 45 °C, and 60 °C, samples were cut to produce semi- and ultrathin sections using an ultramicrotome (Leica UC7, Leica Biosystems, Wetzlar, Germany). Sections were mounted on copper grids (Sigma-Aldrich, St. Louis, MO, USA, 200 mesh) and contrast agents, uranyl acetate 2% and lead citrate, were added. Ultrathin sections were examined using a transmission electron microscope (TEM) HT7700 (Hitachi, Tokyo, Japan) at 100 kV.

### 4.3. Histological and Morphometric Analysis

Counting of myelin fibers and measurement of the cross-sectional area of the sciatic nerve were performed on 1 μm-thick methylene blue-stained sections embedded in Epon. Sections were scanned using an Aperio ImageScope (Leica Biosystems) and analyzed using the manufacturer’s ImageScope software(v12.4.6; Leica Biosystems ImageScope Software Lite). In each cross-section of the peripheral nerve, the total number of myelinated fibers not subject to degradation, as well as the total area occupied by myelinated fibers, was determined.

### 4.4. Intravital Fixation of Nerve Tissues

For immunohistochemical analysis, mice were anesthetized with 6.4% chloral hydrate solution and intracardially perfused with 10% formaldehyde with addition of 0.2% phosphate-buffered glutaraldehyde (GA, Alfa Aesar, Thermo Fisher Scientific, Waltham, MA, USA). After overnight incubation in the same fixative solution, the spinal cord was removed and placed in a 15% sucrose solution for a period of 24–48 h. Before the analysis, the spinal cord was stored in 30% sucrose solution.

### 4.5. Sectioning by Cryotomy

Spinal cord specimens (5 mm long) from the thoracic region were placed in freezing medium (Tissue-Tek O.C.T. Compound, Sakura Finetek, Torrance, CA, USA) at −31 °C for 30 min. Then, 20 µm thick transverse sections were made on a Microm HM 560 cryostat (Thermo Scientific).

### 4.6. Immunofluorescent (IF) Analysis

Blocked with phosphate-buffered saline (PBS) supplemented with 5% goat serum, for 1 h at room temperature, tissue sections were incubated overnight at 4 °C with primary antibodies (PA) (Table 1). Sections were incubated with an appropriate fluorophore-conjugated secondary antibody (Table 1) for 2 h at room temperature. The nucleus was visualized using 4′,6-diamidino-2-phenylindole (DAPI) (10 μg/mL in PBS, Sigma, St. Louis, MO, USA).

### 4.7. Confocal and Light Microscopy

Transverse cuts of the spinal cord (7 μm) were analyzed using a laser confocal microscope Carl Zeiss, LSM 710 META, (Jena, Germany). In each section, an anterior grey horn area was selected to obtain 10 images using 40× objective (APERIOCS2, Leica) and immersion medium 2.6 under the same conditions (laser intensity, magnification, and contrast). Images were used to count glial cells. Each type of cell was identified using specific antibodies summarized in Table 1. Astrocytes were identified as GFAP-positive cells, while NG2 positive cells were oligodendrocyte precursors. Oligodendrocytes were Olig2 antigen positive, and microglia and macrophages had Iba1 marker.

Cell count was carried out using 10 randomly selected fields in the spinal cord. Due to the complex morphology and arrangement of cells, automatic cell counting using programs such as Image Pro is challenging. Two investigators were involved in identification of the cells: first, it was done by a person directly involved in this study followed by a second person not involved in this study. Cell count was completed according to inter-assessment reliability (Williams et a. The ZEN blue Lite version 3.7 software was used for cell counting.

### 4.8. Plasma Collection and Cytokine Analysis

Blood samples were collected into K3EDTA-coated tubes and centrifuged at 2000× *g* for 20 min at 4 °C to obtain plasma. Plasma was aliquoted and stored at −80 °C. Cytokines were analyzed using the Bio-Plex Pro Mouse Cytokine 33-plex Assay (#M60-009RDPD, Bio-Rad, Hercules, CA, USA). The kit included the following analytes: BCA-1, CTACK, ENA-78, Eotaxin, Eotaxin-2, Fractalkine, GM-CSF, I-309, IFN-γ, IL-1β, IL-2, IL-4, IL-6, IL-10, IL-16, IP-10, I-TAC, KC, MCP-1, MCP-3, MCP-5, MDC, MIP-1α, MIP-1β, MIP-2, MIP-3α, MIP-3β, RANTES, SCYB16, TARC, TECK, and TNF-α. Cytokine analysis was completed following the manufacturer’s protocol. Data acquisition was performed using a Luminex 200 analyzer (Merck Millipore, Burlington, MA, USA), and final concentrations were calculated based on standard curves generated for each analyte.

### 4.9. Statistical Analysis

The obtained data were presented as mean ± standard deviation (SD). Normality of data distribution was assessed using the Shapiro–Wilk test, and homogeneity of variances was evaluated using the Brown–Forsythe test. Statistical analysis was performed using one-way ANOVA followed by Tukey’s multiple comparisons test for datasets meeting both normality and variance homogeneity assumptions; otherwise, the non-parametric Kruskal–Wallis test followed by Dunn’s multiple comparisons test was applied. Differences were considered statistically significant at *p* < 0.05. A post hoc statistical power analysis was performed based on the observed sample size, effect sizes, and significance level (α = 0.05). The achieved power for the main comparisons exceeded 0.80, indicating adequate sensitivity to detect the observed effects. Data were analyzed using GraphPad Prism software (GraphPad Software, San Diego, CA, USA; version 8.2.3).

## 5. Conclusions

Our data suggest that the SOD1-G93A transgenic model represents the general phenotype of ALS. However, it appears that this model does not fully reflect the adaptive mechanisms of the autoimmune initiation of the disorder. We found that the early stages of ALS are characterized by ultrastructural changes which are not visible in the light microscope.

We found limited evidence of inflammatory infiltrates in sciatic nerves using immunofluorescence and electron microscopy and electron microscopy, which is consistent with a ‘dying-back’ axonopathy characterized by distal demyelination and fibrosis.

### 5.1. Restriction of the Study

The sample size was relatively small, although this limitation was partially mitigated by the achievement of statistically significant differences. The SOD1-G93A mouse model may not fully recapitulate the autoimmune mechanisms underlying sporadic ALS in humans. Cytokine profiling was restricted to peripheral blood and did not include direct measurements in cerebrospinal fluid or neural tissues.

### 5.2. Limitations of the Study

Specifically, we acknowledge the following limitations:

Single experimental model—the study relied solely on the SOD1-G93A transgenic mouse model, which recapitulates familial ALS associated with SOD1 mutations but may not fully reflect the pathogenic mechanisms of sporadic ALS, particularly autoimmune or non-SOD1-mediated pathways. Limited sample size—although statistically significant differences were achieved, the sample size was relatively small. Discrete time points—analysis was limited to three time points (30, 60, and 90 days), which, while capturing key disease stages, does not allow continuous assessment of disease progression. Peripheral blood cytokine profiling—measurements were performed on peripheral blood and did not include cerebrospinal fluid or neural tissue, which may better reflect local neuroinflammatory processes. Absence of behavioral testing—concurrent behavioral assessment was not performed, precluding direct functional correlation with morphological and molecular changes. Unconfirmed pro-fibrotic phenotype—the pro-fibrotic shift suggested by collagen accumulation on TEM was not confirmed using specific molecular markers.

We also indicate which additional experiments and methods will be used in future studies to address these limitations, namely the following: multiple ALS models (for example TDP-43, FUS transgenic models) to validate findings across different genetic backgrounds and longitudinal behavioral assessment to enable continuous monitoring of disease progression.

## Figures and Tables

**Figure 1:**
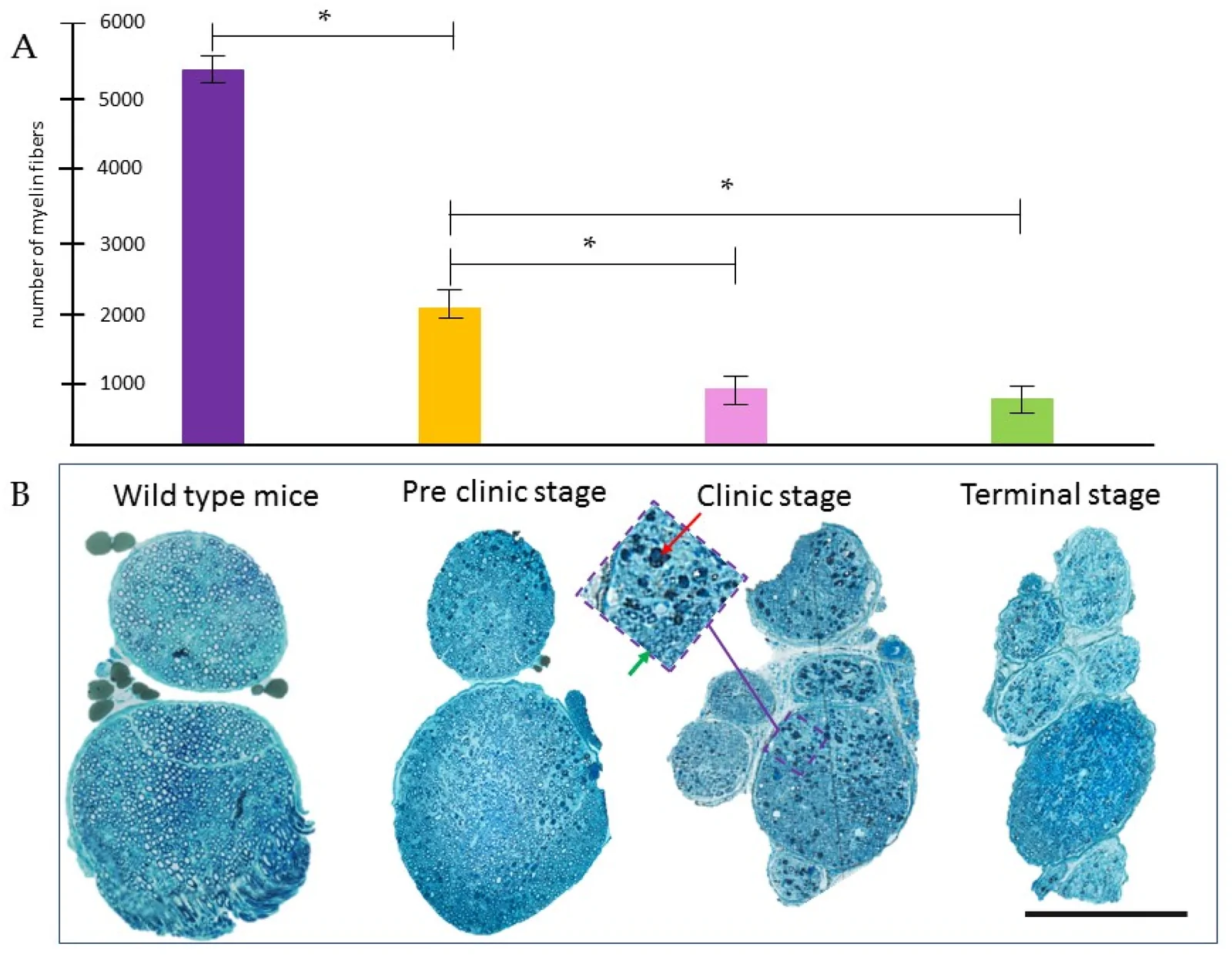
Characteristics of the course of ALS in SOD1-G93A transgenic mice at different stages of the disease. (**A**) Number of myelin fibers. (**B**) Histological structure of the sciatic nerves in the study groups (semi-thin sections, light microscopy). Scale bar: 400 µm. Arrows indicate the following: red—degenerated myelinated fibers; green—intact myelinated fibers *—*p* < 0.05 by ANOVA and following Tukey test analysis of variance, *n* = 5 mice per group.

**Figure 2:**
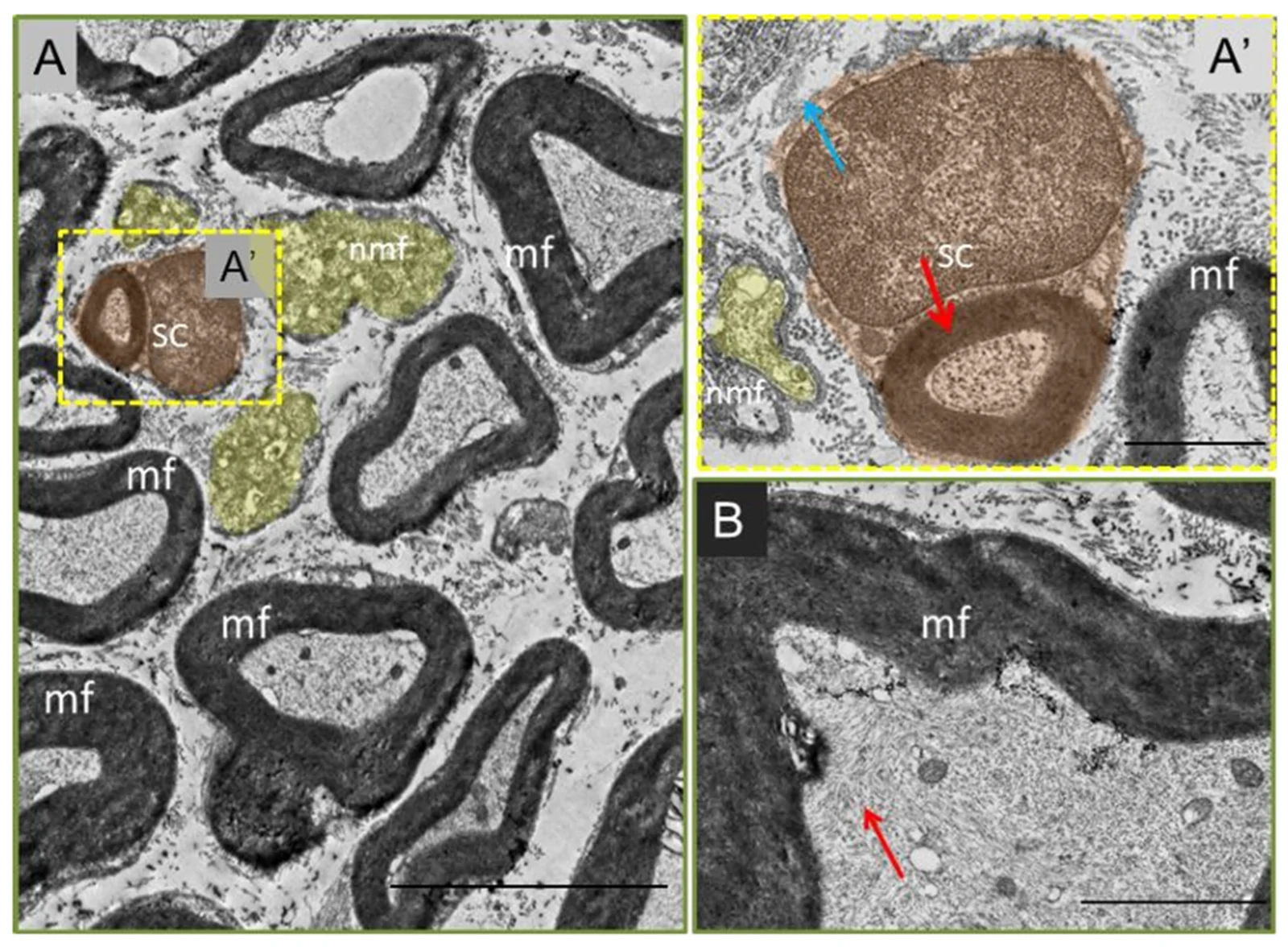
Ultrastructural images of the peripheral nerve of a wild-type mouse. (**A**) General view of the nerve structure containing myelinated fibers (mf), unmyelinated fibers (nmf), and Schwann cells (SC). Scale bar 5 µm; (**A’**) magnified area—Schwann cell ultrastructure (red arrow—clearly visible myelin layers, blue arrow—distinct basement membrane surrounding the cell) (Scale bar 1 µm). (**B**) Ultrastructure of the myelinated fiber and axon, with microfilaments in the axon cytoplasm (shown by the red arrow). Scale bar 2 µm.

**Figure 3:**
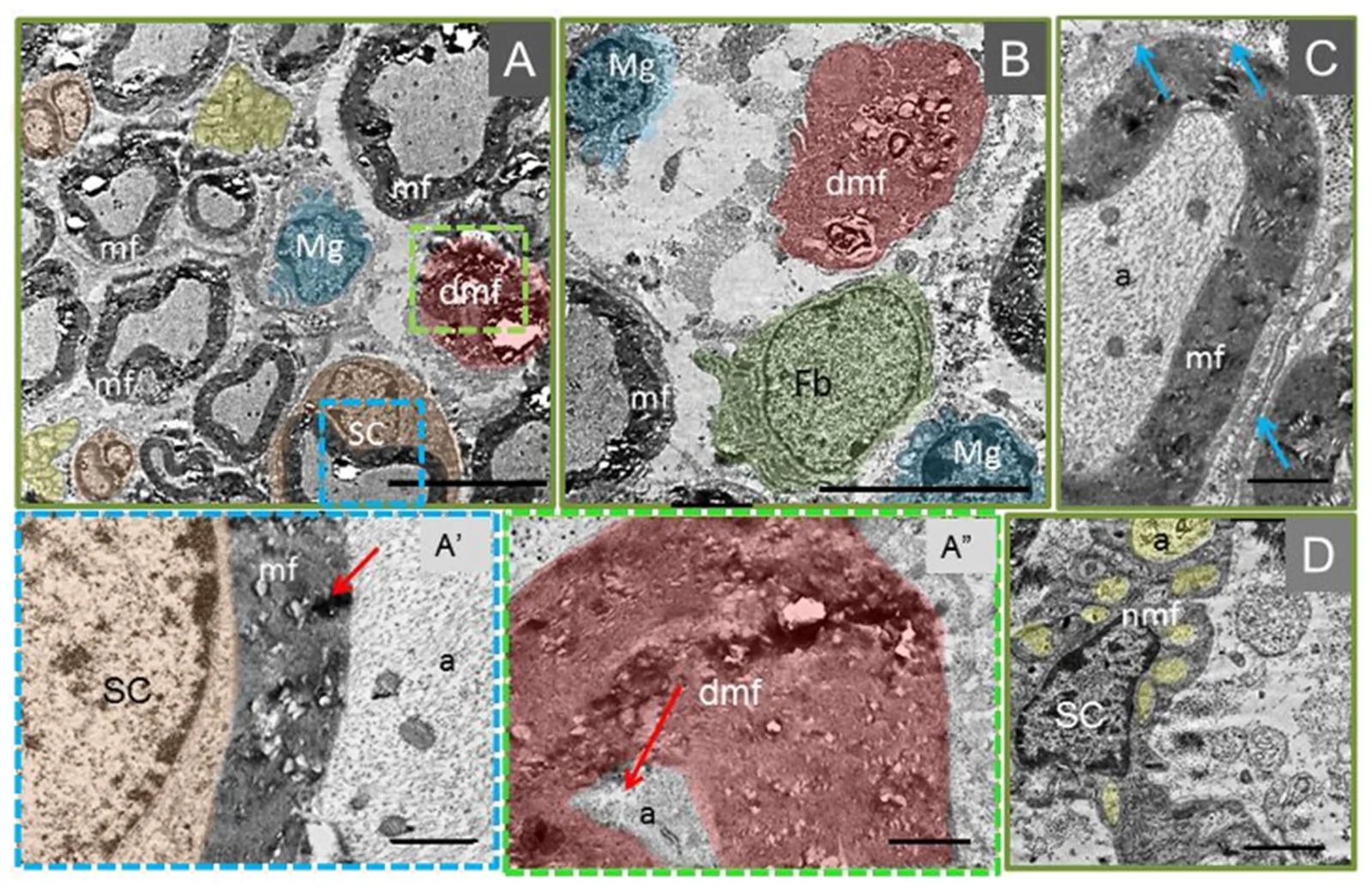
Ultrastructural features of the peripheral nerve of a mouse with ALS at the preclinical stage. (**A**)—overview photograph of a peripheral nerve: the nerve is represented by closely spaced myelinated and unmyelinated fibers marked in yellow, single demyelinated fibers marked in red (according to the hypertrophy type with a myelin index of 4/1, highlighted area (**A’**)). Schwann cells are visualized, marked in orange (highlighted area (**A’**): the cytoplasm of the Schwann cell is completely preserved) and phagocyte cells (microglia) (marked in blue). (**B**)—fibroblasts near the generated myelinated fiber, highlighted in blue. (**C**)—preserved myelinated fiber with a myelin index of 1/5. Arrows indicate preserved basement membranes of myelin-forming Schwann cells. (**D**)—unmyelinated nerve fiber, axons are marked in yellow. Scale bar: (**A**,**B**)—10 µm, (**A’**,**A”**,**C**,**D**)—1 µm. SC—Schwann cell, Fb—fibroblast, Mg—microglial cell, mf—myelinated fiber, dmf—demyelinated fiber, nmf—non-myelinated fiber, a—axon. The myelin index varies. Normally 1/5, with demyelination of the hypertrophy type 4/1. The red marking highlights a demyelinated fiber exhibiting hypertrophic changes with a myelin index of 4/1, indicative of early pathological alterations in the SOD1‑G93A mouse model at the preclinical stage.

**Figure 4:**
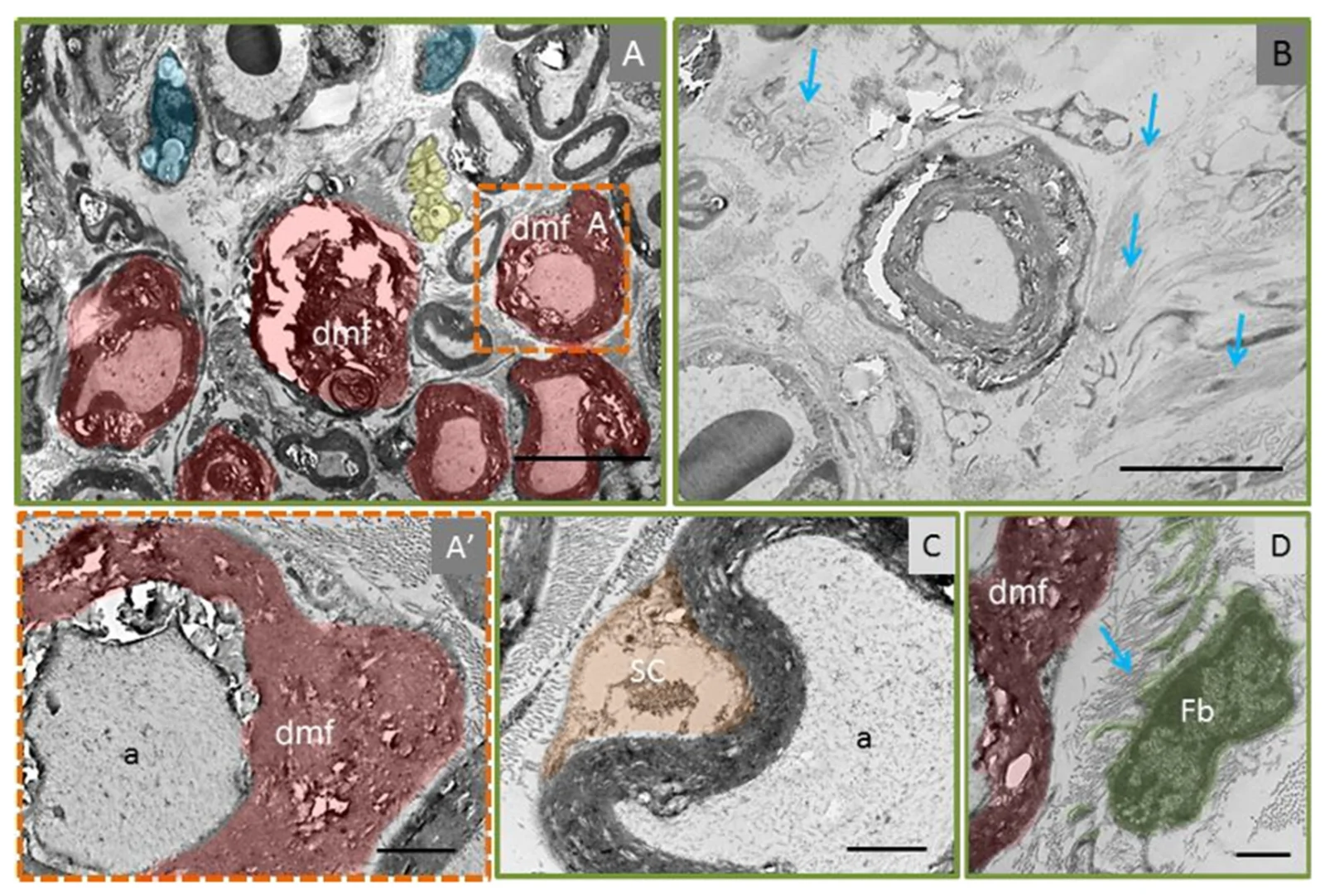
Ultrastructural features of the peripheral nerve of a mouse with ALS at the clinical stage. (**A**)—Overview photo of a peripheral nerve: the nerve is represented by myelinated and unmyelinated fibers (marked in yellow), a large number of demyelinated fibers (marked in red) (according to the type of hypertrophy with a myelin index of 1/1—1/2, in combination with delamination of myelin layers and degeneration of the axial cylinder from the periphery—highlighted area (**A’**)). (**B**)—A large area of the nerve filled with fibrous tissue (collagen fibers, indicated by the arrow) with single degenerating myelin fibers. (**C**)—Schwann cells, marked in orange (highlighted area—the cytoplasm of the Schwann cell is disintegrated, vacuolated). (**D**)—Fibroblasts (marked in green), synthesizing collagen fibers (indicated by the arrow). Scale bar: (**A**)—10 µm, (**B**)—5 µm, (**A’**,**C**,**D**)—1 µm. SC—Schwann cell, Fb—fibroblast, dmf—demyelinated fiber, a—axon.

**Figure 5:**
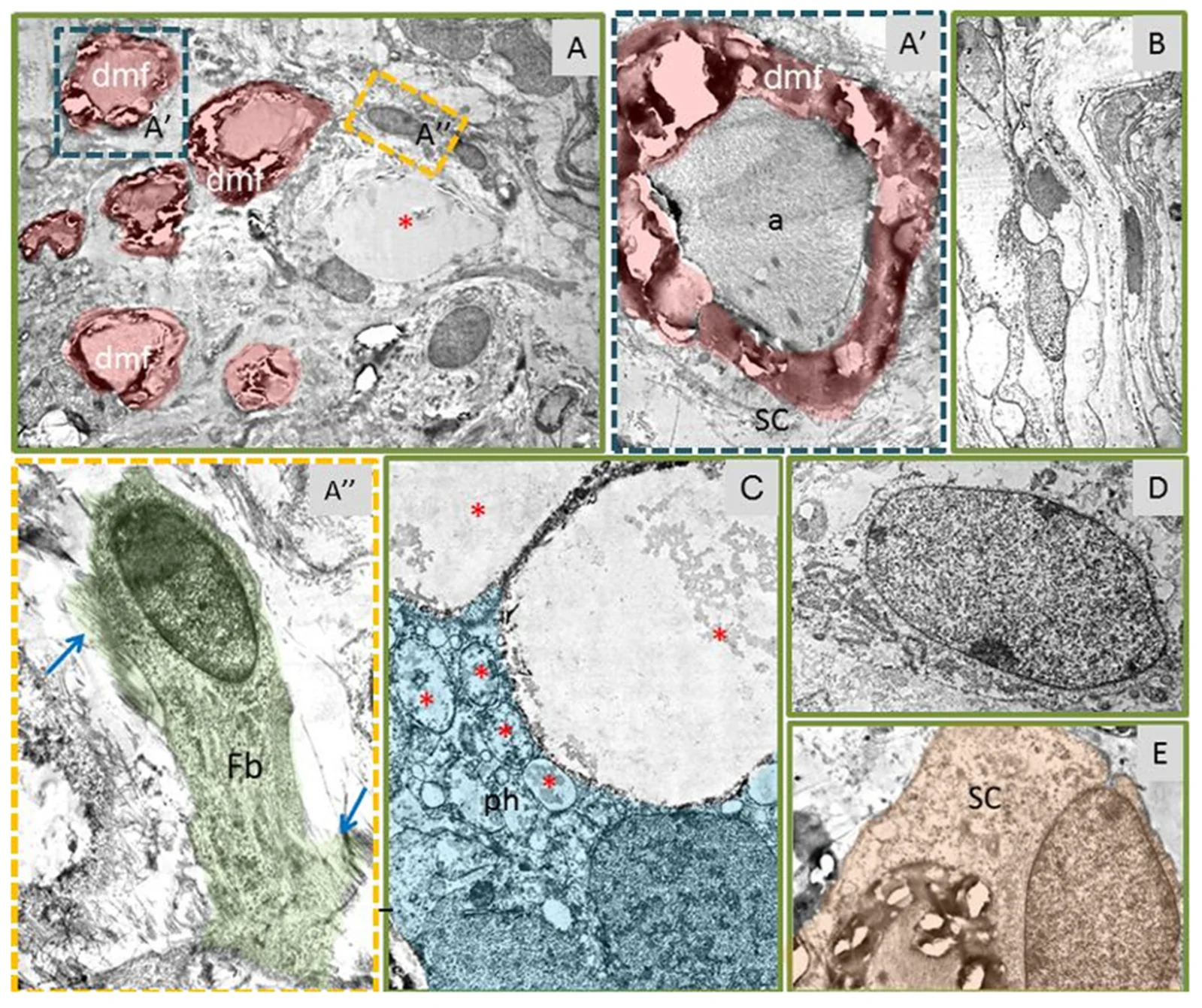
Ultrastructural features of the peripheral nerve structure of a mouse with ALS at the terminal stage. (**A**)—Overview photo of a peripheral nerve: the nerve is replaced by fibrous tissue, among which demyelinating fibers are visualized (marked in red) (according to the type of hypertrophy with a myelin index of 1/1—1/2, in combination with delamination of myelin layers or its complete disintegration in the form of accumulations of osmiophilic material—highlighted area (**A’**)), and extensive areas of fibrous tissue and fibroblasts. (**A”**)—Fibroblasts (marked in green), synthesizing collagen fibers (indicated by an arrow). (**B**)—Epineurium, showing a hypertrophied ultrastructure and multilayeredness; (**C**)—A phagocyte cell, in the cytoplasm of which there are numerous large and small autophagic vasculature (marked with an asterisk). (**D**)—A regenerating fibroblast. (**E**)—Schwann cells, marked in orange (the highlighted area is the cytoplasm of a Schwann cell disintegrated). Scale bar: (**A**)—10 µm, (**B**)—5 µm, (**A’**,**C**,**D**)—1 µm. SC—Schwann cell, Fb—fibroblast, dmf—demyelinated fiber, a—axon.

**Figure 6:**
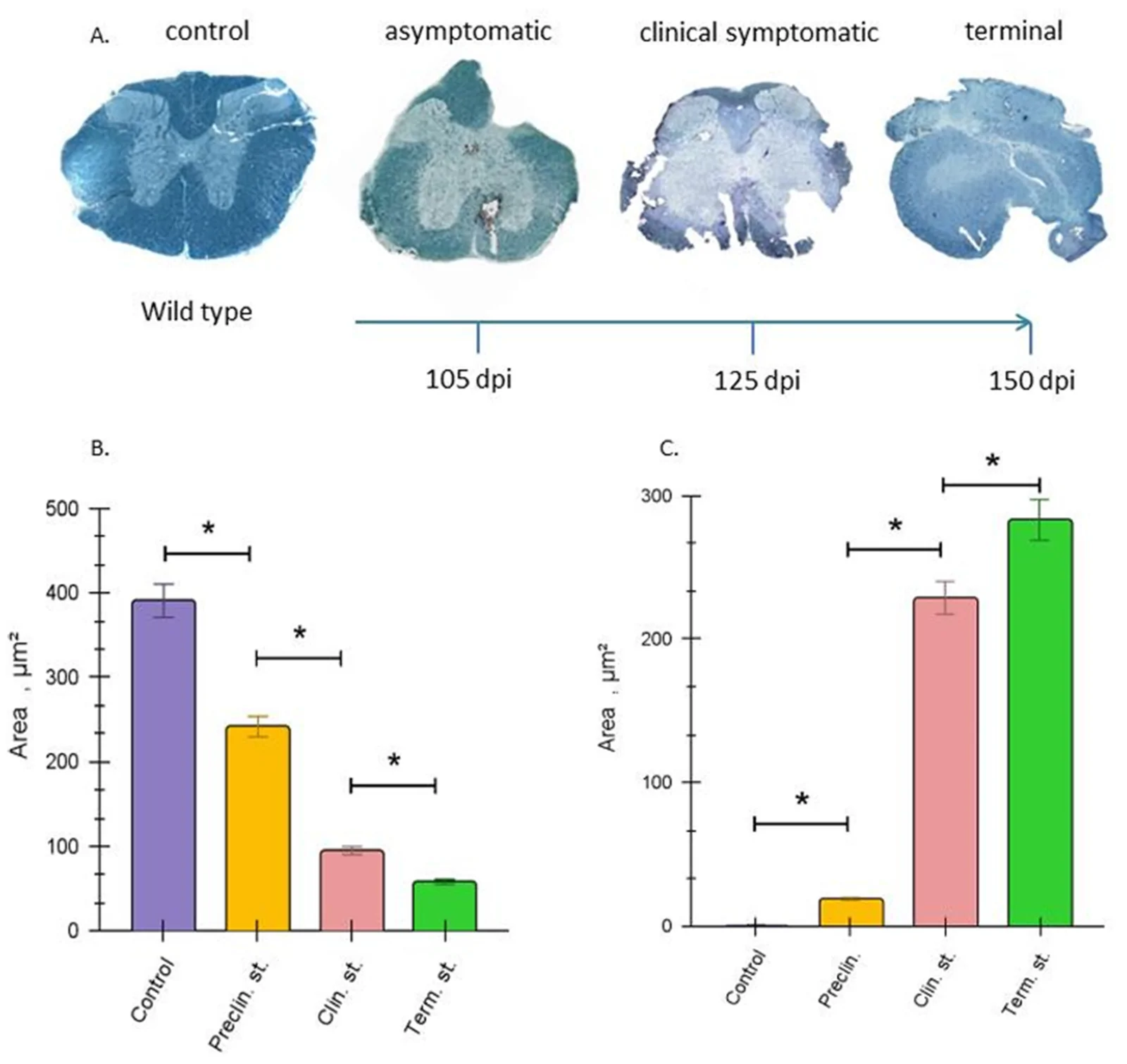
Damage in a spinal cord at different stages of ALS development: (**A**) Transverse semithin sections of the spinal cord at the level of the lumbar enlargement, stained with toluidine blue. Scale bar: (**B**) Area of intact spinal cord tissue; (**C**) Area of damaged and degenerated spinal cord tissue. *—*p* < 0.05 by one way analysis of variance, n = 5 rats per group.

**Figure 7:**
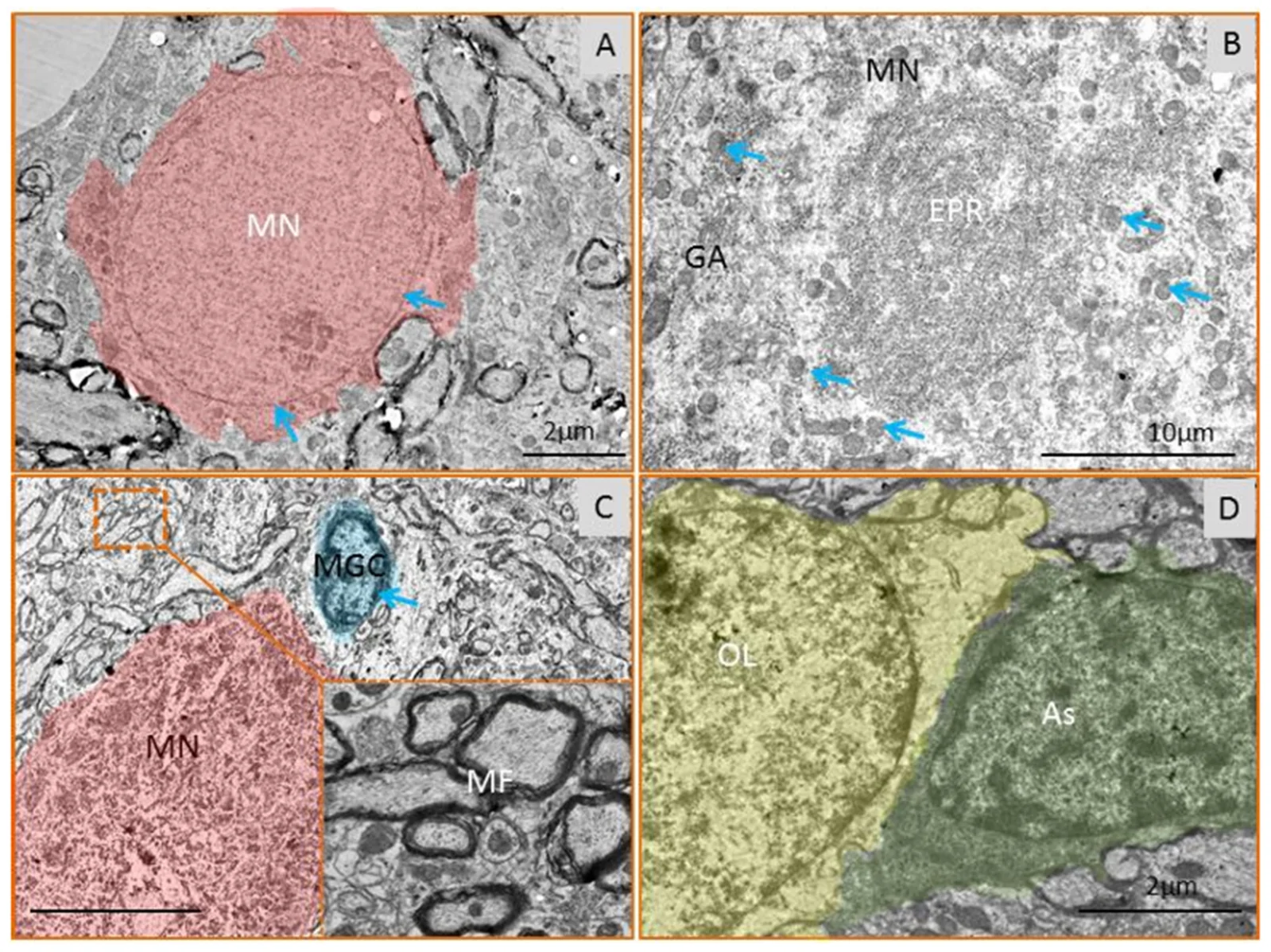
Ultrastructure of the gray matter of the anterior horn of the spinal cord of a wild-type mouse. (**A**) General structure of a normal functioning motor neuron (MN, highlighted in red) without signs of degeneration with a rounded nucleus and a clear karyolemma (arrow). (**B**) Ultrastructure of the cytoplasm of a motor neuron (MN, highlighted in red): well-developed cisterns of the rough ER (EPR), Golgi complex (GA), a large number of rounded mitochondria with clearly developed cristae (arrow). (**C**) Normal motor neuron surrounded by myelinated fibers (in the enlarged area the structure of myelinated fibers with clear myelin layers, MF), and an inactivated microglial cell (MGC, highlighted in blue) 2 µm in diameter with an oval-shaped nucleus, a tortuous karyolemma containing heterochromatin (arrow). (**D**)—Non-activated astrocyte (As, highlighted in green) 4 µm in diameter with an irregularly shaped nucleus containing heterochromatin and a normal oligodendrocyte with a strictly oval-shaped nucleus and electron-clear cytoplasm (highlighted in yellow). Scale bar: (**A**,**D**)—2 µm, (**B**)—10 µm, (**C**)—5 µm.

**Figure 8:**
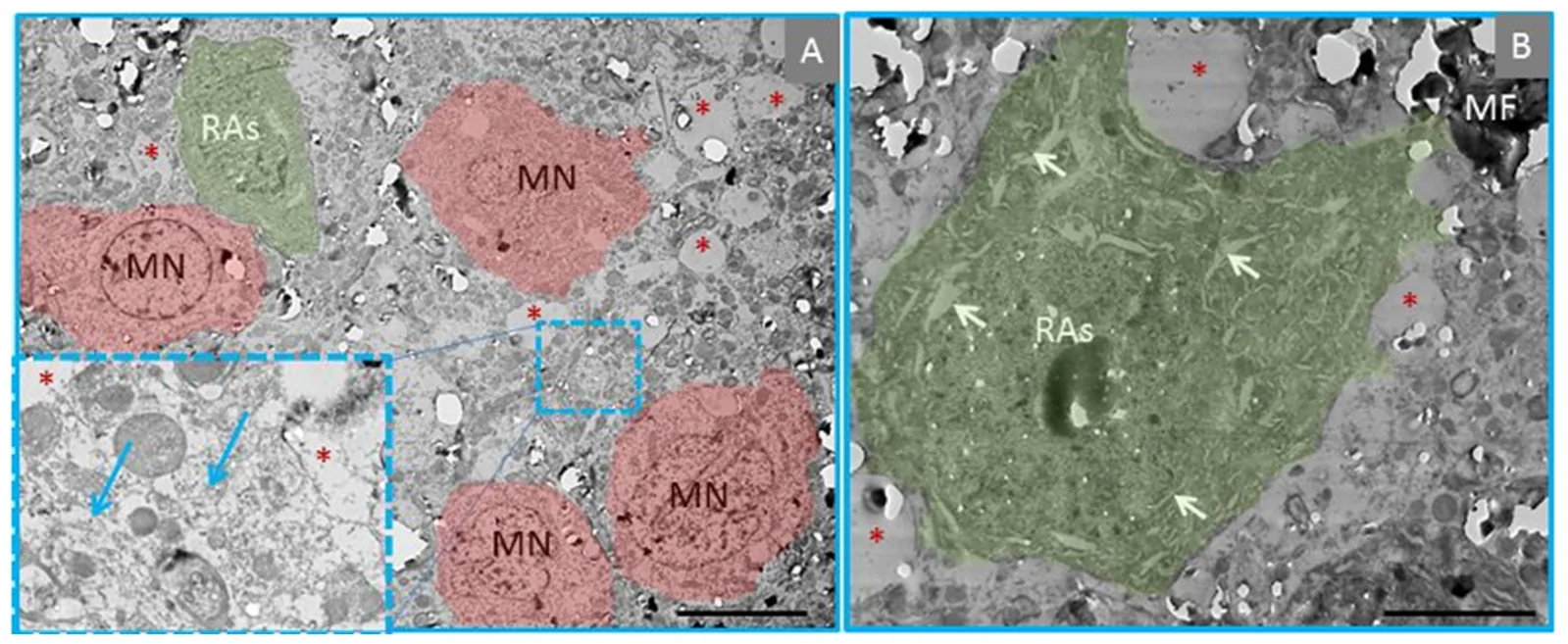
Ultrastructure of gray matter of anterior horns of mouse spinal cord with SOD1 model of ALS at the stage of preclinical manifestations. (**A**)—General view: among partially disintegrated (arrow) or completely degenerated and represented by enlightened areas (*) of nerve fibers (enlarged area), motor neurons are visualized (MNb are highlighted in red) with signs of necroptosis (absence of membrane, cavities in the cytoplasm) and reactive astrocytes (Ras, highlighted in green). (**B**)—Ultrastructure of reactive astrocyte of irregular pyramidal shape with electron-dense cytoplasm, well-developed endoplasmic reticulum (arrow), 18 μm in diameter (Ras, highlighted in green). Scale segment: (**A**)—10 µm, (**B**)—5 µm.

**Figure 9:**
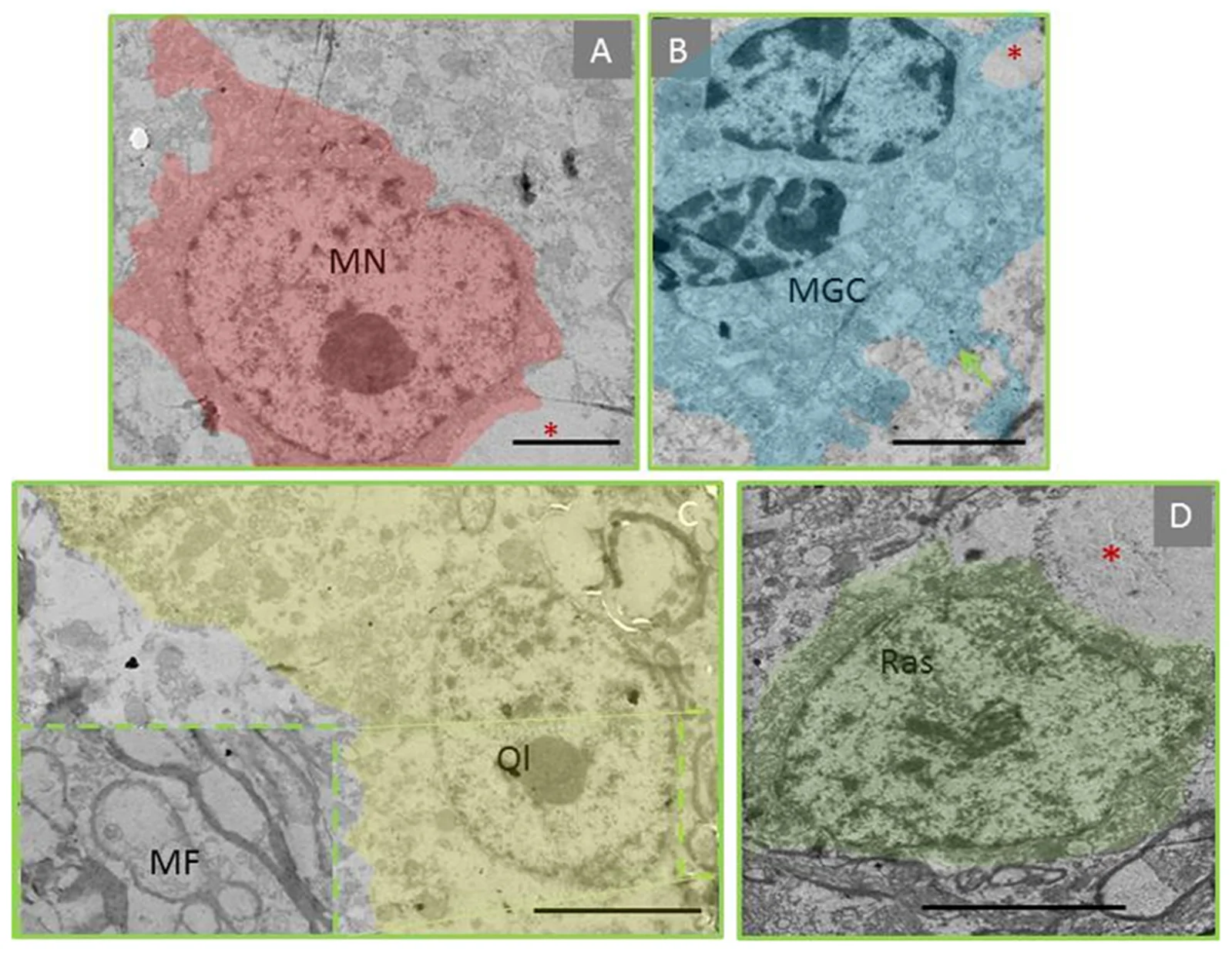
Ultrastructure of the gray matter in the anterior horns of the spinal cord of a mouse SOD1 model of ALS at the stage of clinical manifestations. (**A**) Ultrastructure of a motor neuron (MN, highlighted in red) with disintegrated cytoplasm among completely degenerated nerve fibers represented by clear areas (*). (**B**) Ultrastructure of activated microglial cells (MGC, highlighted in blue). Irregularly shaped cytoplasmic processes of the cells with medium-density cytoplasm surround the clear areas and are in close contact with partially disintegrated fibers (arrow). (**C**) Degenerating oligodendrocyte (Ol, highlighted in yellow) with clear cytoplasm and adjacent myelinated fibers (MF) (enlarged area) with signs of degeneration (myelin layers are not identifiable). (**D**) Ractive astrocytes (Ras, highlighted in green), degenerated nerve fibers represented by clear areas (*). Scale bar: (**A**)—10 µm, (**B**,**C**)—5 µm. (**D**)—20 µm.

**Figure 10:**
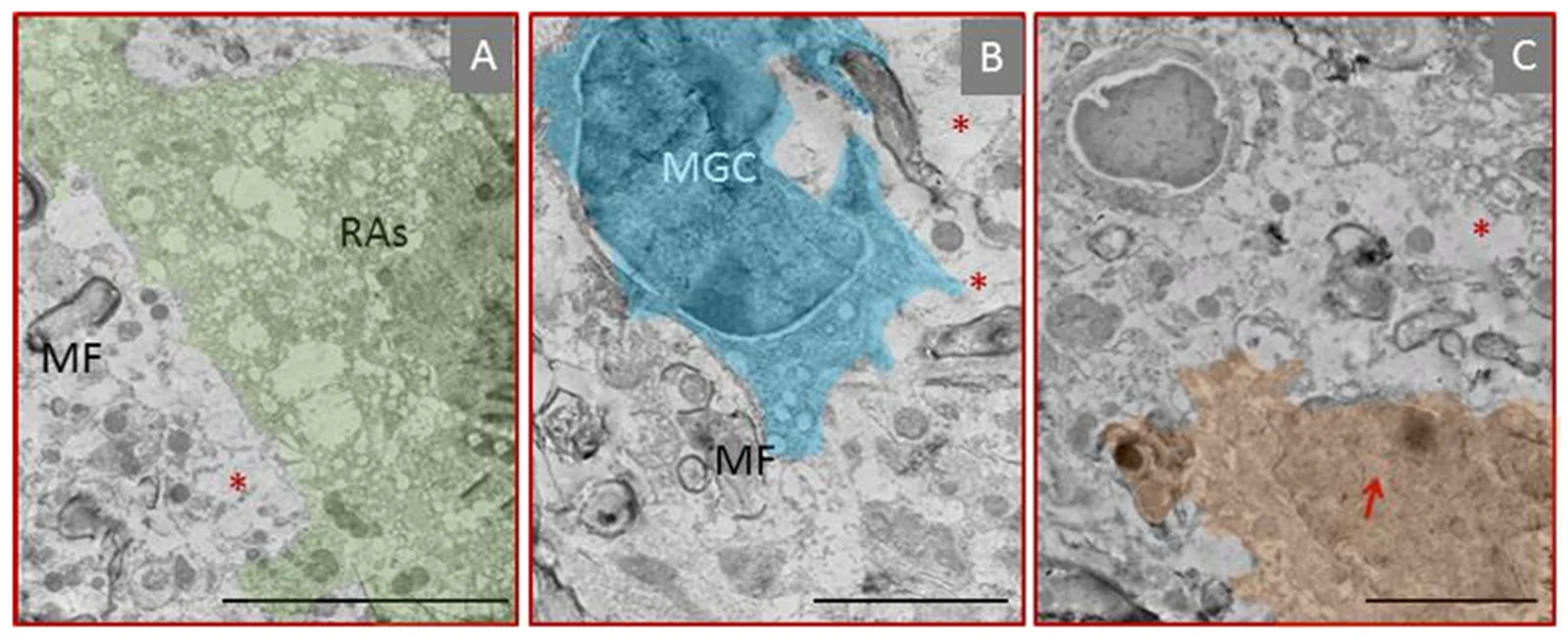
Ultrastructure of the gray matter in the anterior horns of the spinal cord of a mouse SOD1 model of ALS at the terminal stage of the disease development. (**A**)—General view of the state of disintegrated gray matter (arrow), which appears electron-transparent; reactive astrocytes (Ras, highlighted in green) with swollen mitochondria with a cleared matrix are identified. (**B**)—Ultrastructure of activated microglial cells (MGC, highlighted in blue) and individual demyelinated fibers among extensive cleared areas without any structures (*). (**C**)—Cells of unknown genesis, possibly NG2-glial cells (arrow, highlighted in orange). Scale bar: (**A**)—20 µm, (**B**,**C**)—10 µm.

**Figure 11:**
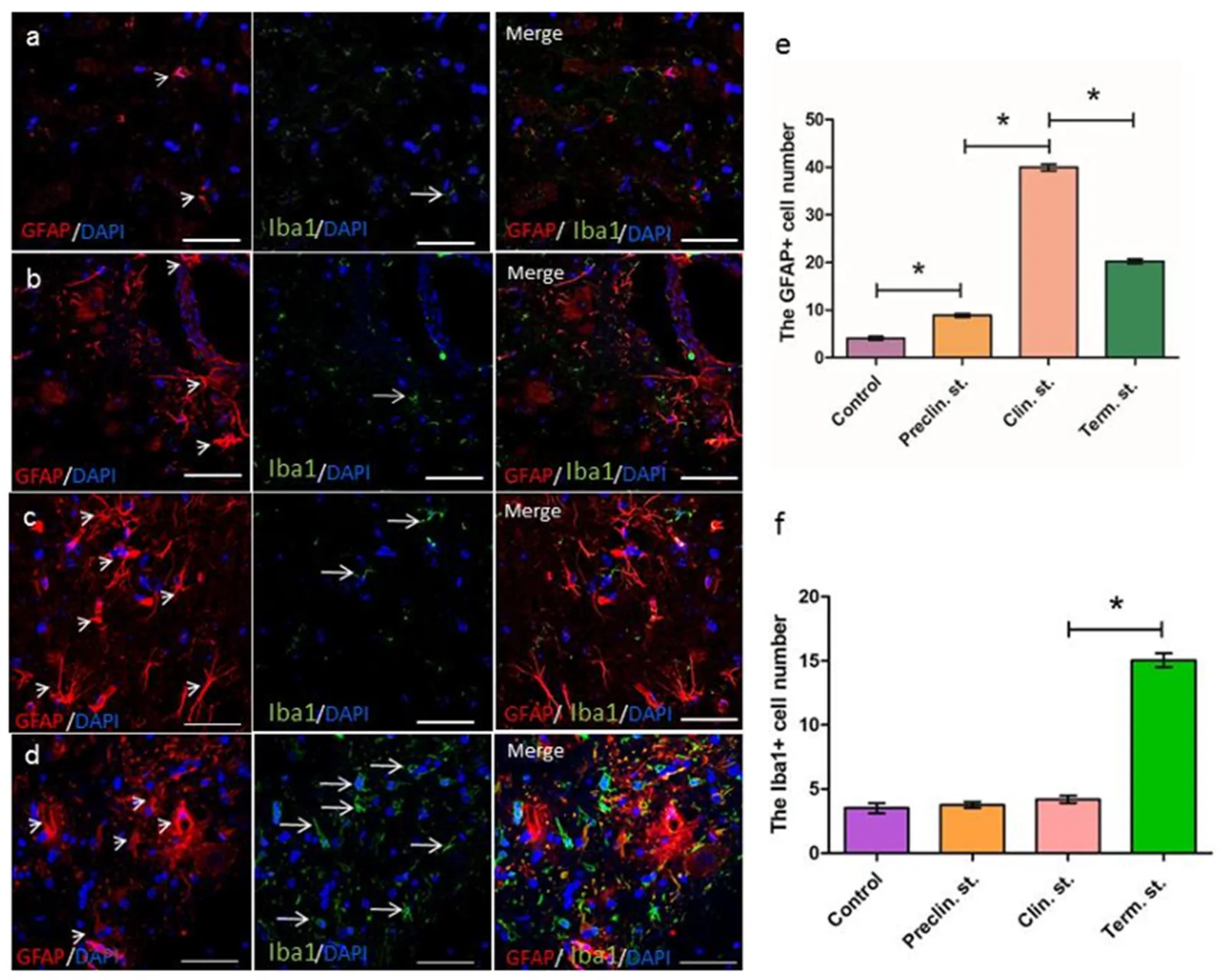
Double immunohistochemical staining for glial acidic fibrillary protein (GFAP) (red) and Allograft inflammatory factor 1 (AIF-1)—microglia marker Iba1 (green) in the ventral horn of gray matter of mouse spinal cords in cross section at the T8 vertebra level. (**a**)—In wild-type mice; and with SOD1—ALS model at (**b**)—presymptomatic, (**c**)—symptomatic, (**d**)—terminal stages of disease progression. Nuclei were counterstained with 4′,6-diamidino-2-phenylindole (DAPI; blue). Scale bars = 50 μm. Arrowheads indicate GFAP positive astrocytic processes. Arrows indicate Iba1 positive microglial cells processes. (**e**,**f**)—Quantification of different glial cell types as the disease progresses and in wild-type mice. Abscissa axis—intact control and disease stages (n = 3), ordinate axis—number of cells: E—GFAP+ astrocytes; F—Iba1+ microglial cells, *—*p* < 0.05 by ANOVA and following Tukey test analysis of variance, n = 5 mice per group.

**Figure 12:**
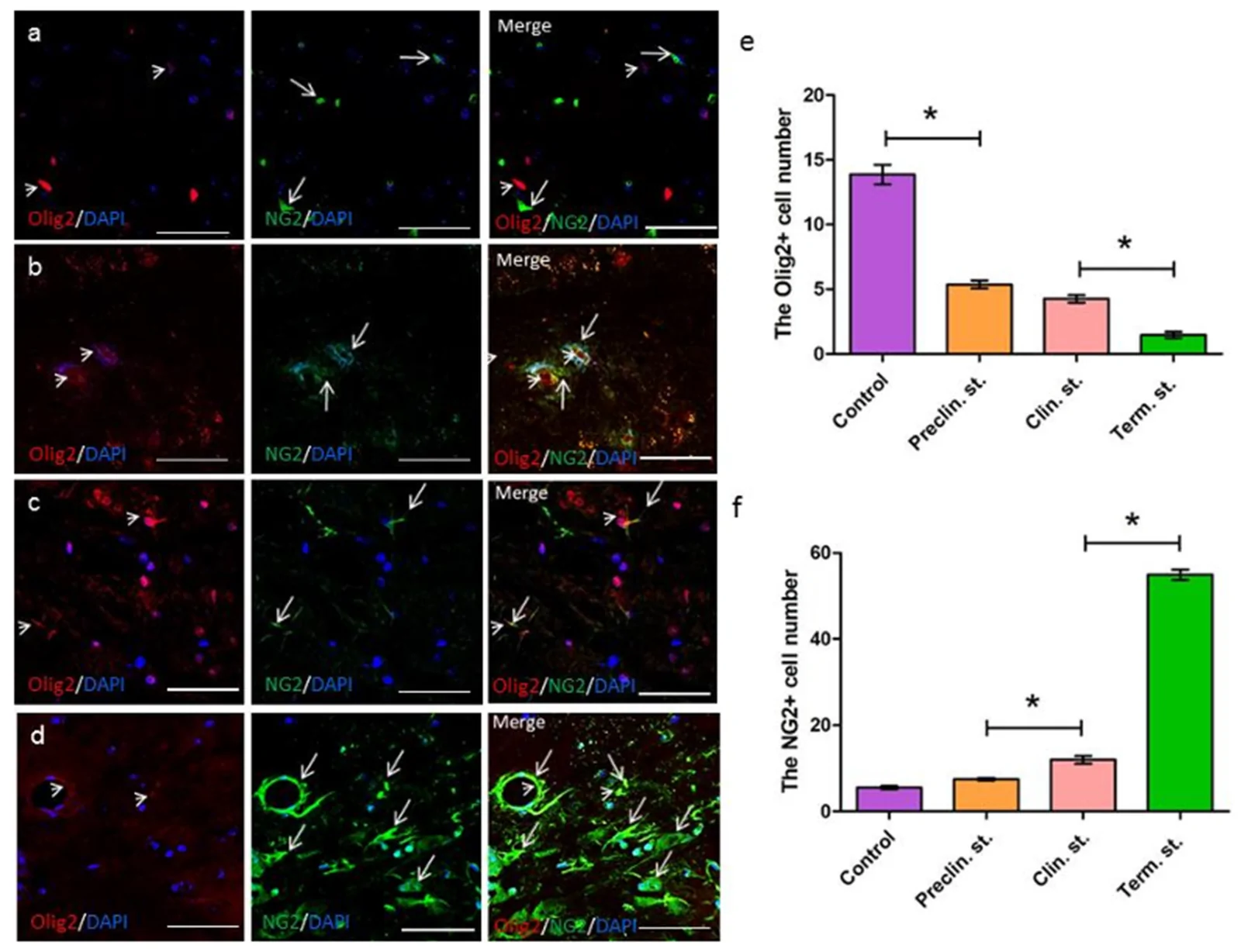
Double immunohistochemical staining for oligodendrocyte transcription factor 2 (OLIG2) (red), an oligodendrocyte marker, and chondroitin sulfate proteoglycan (NG2) (green) in the ventral horn of the gray matter of mouse spinal cords in a cross section at the T8 vertebra. (**a**)—In wild-type mice; and with SOD1—an ALS model at (**b**)—presymptomatic, (**c**)—symptomatic, (**d**)—terminal stages of disease progression. Nuclei were counterstained with 4′,6-diamidino-2-phenylindole (DAPI; blue). Scale bars = 50 μm. Arrowheads indicate OLIG2-positive oligodendrocite nucleus. Arrows indicate NG2-positive cells and processes. (**e**,**f**)—Quantification of different glial cell types as the disease progresses and in wild-type mice. Abscissa axis—intact control and disease stages (n = 3), ordinate axis—number of cells—Olig2+ oligodendrocytes, NG2+ Ng2-glial cells. *—*p* < 0.05 by ANOVA and following Tukey test analysis of variance, n = 5 mice per group.

**Figure 13:**
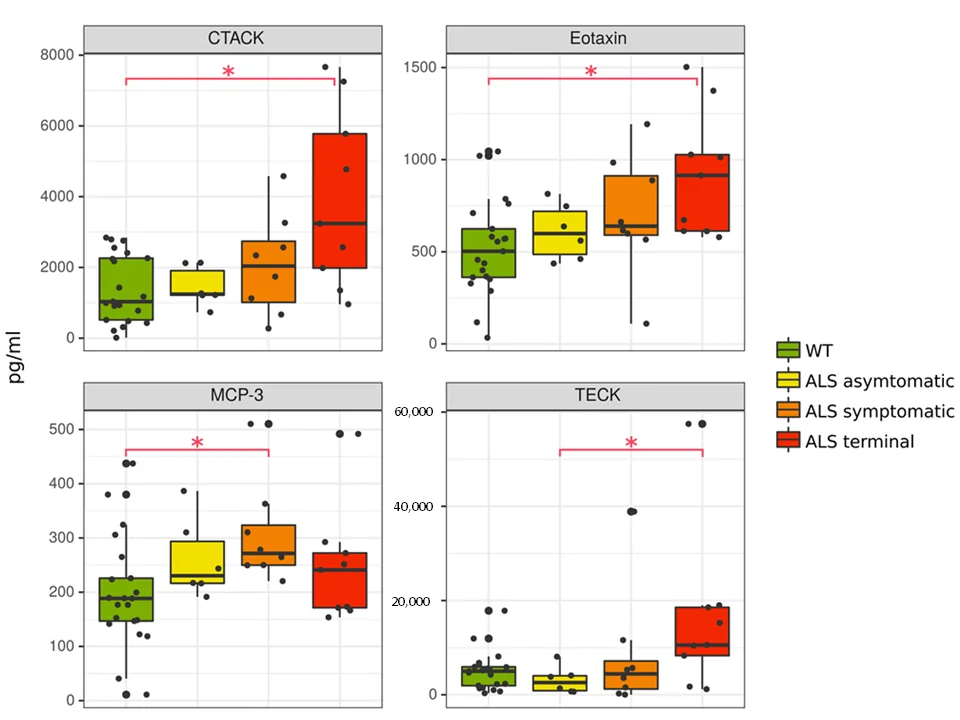
Significant differences in serum cytokine levels in transgenic SOD1-G93A mice expressing the amyotrophic lateral sclerosis phenotype. ALS asympt and WT mice do not differ. ALS symptom has elevated MCP-3 compared to WT. ALS term has elevated CTACK and Eotaxin compared to WT. ALS asympt and ALS symptom do not differ. ALS term has elevated TECK compared to ALS asympt. Symptom and ALS term do not differ. Statistical analysis performed using Kruskal–Wallis test with Dunn’s post hoc analysis, *—*p* < 0.05, WT *n* = 21 mice, ALS asymptomatic *n* = 6 mice, ALS symptomatic *n* = 8 mice, ALS terminal *n* = 9 mice.

**Figure 14:**
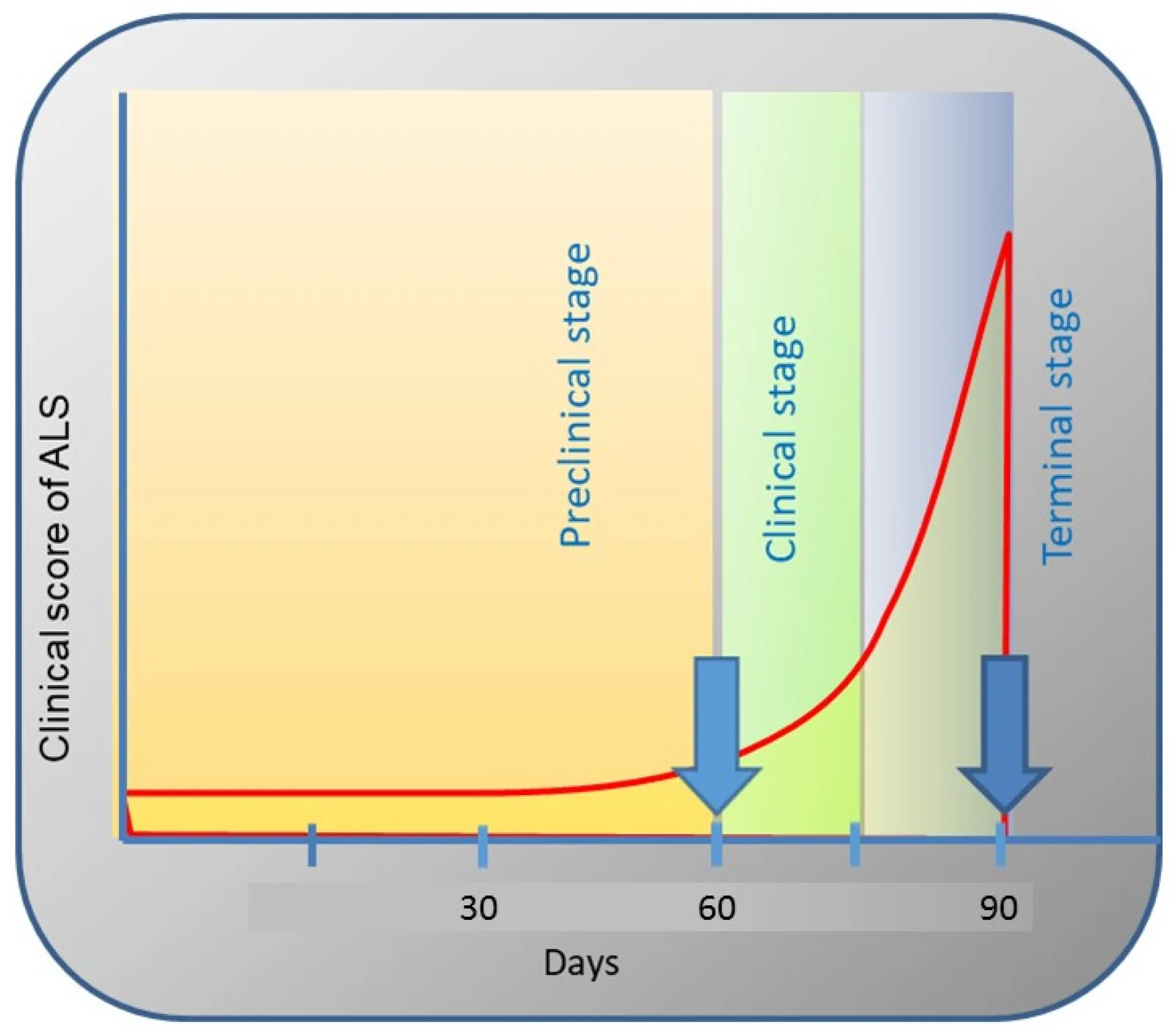
Characteristics of the course of ALS in SOD1 transgenic mice at different stages of the disease clinical manifestations.

**Table 1:** Primary and secondary antibodies used in IF analysis.

Antigen	Species	Dilution	Source
Iba1	Rabbit	1:200	Abcam
Glial fibrillary acidic protein (GFAP)	Mouse	1:200	Santa Cruz
Olig-2	Mouse	1:200	Santa Cruz
NG2	Rabbit	1:100	Abcam
Anti-mouse IgG conjugated to Alexa 488	Donkey	1:200	Invitrogen
Anti-rabbit IgG conjugated to Alexa 647	Donkey	1:200	Invitrogen

## Data Availability

All data generated or analyzed during this study are included in this published article. The data that support the findings of this study are available from the corresponding author upon request.

## Supplementary Materials

The following supporting information can be downloaded at: https://www.mdpi.com/article/10.3390/ijms27198604/s1.
