# Supplementary material for: Phenotypic Profiling of SOD1-G93A Mice: Integrating Sciatic Nerve Histopathology with Systemic Chemokine Dynamics During ALS Progression
Source: Int J Mol Sci. 2026 Sep 25;27(19):8604. doi: 10.3390/ijms27198604 (PMC13665082; doi:10.3390/ijms27198604)
Supplement: Supplementary file 1 [file ijms-27-08604-s001.zip › ijms-4480048-supplementary.pdf]

id	group	BCA-1	CTACK	ENA-78	Eotaxin	Eotaxin-2	Fractalkine
1	ALS asypmtomatic	44681,19	1266,48	1048,49	637,42	5990,59	101,97
2	ALS asypmtomatic	2284,67	1213,66	15515,45	560,4	6185,52	109,43
3	ALS asypmtomatic	4028,87	1221,62	12345,57	747,01	14023,42	233,8
4	ALS asypmtomatic	11988,55	733,7	35373,94	460,96	3872,82	72,2
5	ALS asypmtomatic	21871,81	2122,59	4657,23	436,27	11090,9	277,85
6	ALS asypmtomatic	6317,77	2132,94	12062,62	814,21	5278,15	135,97
7	ALS sypmtomatic	29978,38	3260,84	3942,18	616,35	10723,5	450,79
8	ALS sypmtomatic	19167,674	2339,44	23583,18	984,23	8095,43	120
9	ALS sypmtomatic	30608,51	273,75	3111,6	109,74	2310,36	71,11
10	ALS sypmtomatic	26801,16	1738,36	1417,94	598,39	6572,5	268,08
11	ALS sypmtomatic	5449,47	668,92	12909,01	566,35	8051,84	126,4
12	ALS sypmtomatic	24161,35	4579,7	37038,8	1192,73	7423,56	326,14
13	ALS sypmtomatic	10187,42	1126,85	10497,49	661,98	5707,55	163,91
14	ALS sypmtomatic	24345,74	2565,79	2618,54	888,07	9453,85	102,54
15	ALS terminal	19760,51	4775,32	3885,44	914,59	5110,32	187,94
16	ALS terminal	27356,01	7664,66	5686,25	1012,17	3016,02	147,51
17	ALS terminal	23916,26	1982,74	4882,44	612,01	4370,98	293,27
18	ALS terminal	4349,45	958,84	36249,26	672,56	8443,32	59,02
19	ALS terminal	13815,27	1350,8	14304,53	579,55	5769,84	125,81
20	ALS terminal	16512,65	5777,81	9921,97	1374,14	12654,96	319,2
21	ALS terminal	22776,68	3242,18	16851,45	1027,3	7793,77	430,69
22	ALS terminal	32059,58	7253,46	13343,76	1502,93	9187,19	531,67
23	ALS terminal	13772,01	2571,01	7962,46	612,84	4749,84	281,79
24	WT	16567,72	313,7	7298,88	399,54	1874,34	50,43
25	WT	11327,82	2757,02	8203,15	1044,72	12164,74	130,57
26	WT	33644,39	2842,57	66055,77	571,68	6167,08	161,74
27	WT	9510,2	912,66	5387,75	437,57	4490,7	136,99
28	WT	28697,92	2791,37	16074,45	351,4	5418,78	216,45
29	WT	2242,09	13,59	66,27	33,46	332,6	130,44
30	WT	8321,46	2262,09	3895,34	710,27	6015,25	161,14
31	WT	2625,06	483,93	12651,1	502,77	6797,87	146,29
32	WT	3937,27	516,75	2343,29	456,18	9563,24	118,15
33	WT	6676,05	776,77	12909,01	581,7	3825,66	122,85
34	WT	17002,31	429,04	3760,39	569,88	3913,13	179,22
35	WT	28596,07	2555,52	2024,17	1020,92	8723,47	136,57
36	WT	17756,98	1429,32	1445,96	786,79	4485,6	160,16
37	WT	21851,95	1033,01	2745,8	760,6	3302,47	142,26
38	WT	8788,15	933,67	1349,37	287,6	963,44	73,26
39	WT	16157,57	2410,62	3925,12	327,85	2386,75	111,24
40	WT	9319,26	2172,51	1576,77	365,82	3267,83	199,95
41	WT	2279,86	210,28	531,38	117,18	749,83	19,48
42	WT	13788,32	2241,43	574,24	624,37	3861,19	97,92
43	WT	7581,02	1174,53	958,88	555,38	2895,25	95,28
44	WT	7256,57	994,22	893,78	361,35	5494,34	149,75

GM-CSF	I-309	IFN-g	IL-1b	IL-2	IL-4	IL-6	IL-10	IL-16
7,91	48,07	15,29	205,42	13,37	3,38	13,68	273,27	709,49
9,34	24,06	17,09	1119,88	10,03	2,98	11,43	200,79	1084,05
18,29	26,5	51,13	412,66	23,29	5,18	21,75	263,4	1874,67
2,74	23,08	12,38	205,42	14,27	3,28	8,26	136,32	1091,75
14,74	23,14	29,82	1772,43	42,78	17,66	99,04	544,92	1271,64
14,25	56,27	20,08	1032,18	10,95	12,87	57,44	260,61	1348,79
33,39	30,76	34,3	492,02	33,32	20,08	71,88	1188,81	953,85
20,53	8,66	13,51	320	47,34	7,66	41,31	148,89	639,11
2,74	10,14	10,33	142,06	5,34	1,62	18,68	57,29	2480,97
55,64	46,41	45,72	416,83	46,77	9,67	63,46	558,93	512,05
8,27	21,98	17,09	207,23	5,98	3,05	15,83	138,43	1803,75
52,31	32,84	89,42	422,86	48,16	9,38	376,08	363,39	1498,3
1,38	29,37	22,02	443	17,76	5,22	18,63	255,33	1155,33
8,09	32,66	10,96	221,71	41,07	3,09	24,1	267,86	473,65
27,08	58,96	28,95	271,97	40,78	6,92	34,03	462,55	878,19
13,43	34,98	18,58	280,89	21,4	6,78	54,52	415,38	823,87
12,76	14,65	52,54	420,33	32,74	8,16	80,64	623,52	1420,65
2,74	42,65	11,19	228,93	4,38	1,9	5,75	96,6	1546,75
12,76	51,03	13,18	206,33	8,95	3,49	29,95	245,98	407,92
43,66	47,96	96,39	474,62	45,9	11,45	42,04	549,03	1310,98
46,22	24,6	72,25	450,97	53,16	12,9	32,33	695,12	1089,37
46,59	41,12	86,22	437,99	46,47	15,75	61,28	704,58	1485,83
33,03	50,55	54,37	399,28	32,6	10,87	69,12	488,01	407,94
5,16	4,16	18,84	203,45	17,76	3,15	9,58	77,03	87,55
16,66	19,35	44,67	452,96	21,26	5,35	30,67	279,34	863,47
10,95	41,94	35,65	395,13	56,43	7,1	36,82	283,29	2086,04
9,6	54,61	36,5	308,46	171,33	3,76	14,3	156,73	1561,8
9,69	9,31	22,77	929,03	34,04	10,85	70,35	588,3	737,72
2,74	3,72	15,81	1,01	32,725	4,25	23,14	27,31	44,59
23,64	66,7	24,99	251,4	14,88	5,55	47,23	373,84	731,38
9,69	22,61	15,61	245,12	6,61	2,79	16,36	189,13	1380,4
2,74	2,49	10,17	185,39	13,07	1,83	2,99	127,77	1804,81
17,93	20,62	19,33	216,29	9,41	4,12	23,38	140,54	811,92
43,98	10,27	31,4	168,89	14,58	5,94	29,84	208,48	701,88
12,09	29,62	23,96	279,11	31,88	5,66	22,41	208,48	728,72
34,77	106,15	41,89	287,26	39,57	5,48	29,48	349,42	752,87
15,63	75,63	36,71	226,92	41,4	6,88	27,95	271,27	1093,47
14,47	15,57	15,72	109,97	35,2	4,13	13,23	274,18	282,89
17,19	61,36	19,42	185,35	41,4	11,36	27,85	309,02	426,34
37,96	32,66	34,07	251,31	52,96	10,31	37,06	527,37	942,08
8,38	21,03	11,42	96,78	39,11	1,26	3,92	56,4	98,72
11,97	42,96	14,79	221,45	32,61	4,97	17,33	176,57	536,69
20,41	21,97	20,12	189,57	26,16	6,04	25,4	153,35	447,86
28,79	47,43	22,4	195,17	33,59	5,65	21,13	202,79	368,32

IP-10	I-TAC	KC	MCP-1	MCP-3	MCP-5	MDC	MIP-1a	MIP-1b
363,03	146,02	33,53	266,98	243,57	424,31	173,01	7,2	56,91
353,93	113,26	22,88	215,19	217,02	178,61	142,53	5,12	62,54
805,25	265,94	168,56	459,09	216,22	207,37	124,49	11,14	103,82
289,54	83,65	8,46	159,32	310,33	65,94	120,63	6,84	43,14
465,08	263,01	67,8	453,44	386,61	53,09	323,6	16,24	73,47
535,65	100	39,63	329,9	191,4	199,59	286,94	16,46	77
626,48	367,2	106,46	460,47	363,01	97,98	229,17	33,93	106,53
560	91,6	49,33	308,35	250	190,76	190,85	16	52,48
233,85	57,01	28,01	87,96	310,43	63,31	119,09	1,87	29,78
562,22	301,4	60,66	550,75	264,6	372,35	202,21	27,07	170,17
359,09	111,39	31,63	237,68	249,54	232,69	162,82	6,65	55,18
981,44	579,48	583,63	826,62	510,25	282,33	309,27	26,86	207,96
609,95	271,86	38,73	271,34	278,44	170,18	176,63	11,08	33,53
389,8	160,59	47,78	261,73	220,42	230,73	215,72	10,03	57,75
479,79	273,53	53,62	462,79	166,35	391,81	274,1	22,24	105,05
387,7	228,91	60,36	283,83	171,32	55,92	181,61	14,59	75,73
481,59	240,7	14,24	526,93	491,92	144,46	250,34	26,51	75,73
345,09	63,87	28,01	129	153,62	194,21	89,48	2,58	40,69
341,72	148,8	31,57	242,49	272,24	302,38	130,87	7,26	69,78
1064,82	528,59	66,71	736,1	251,52	168,71	470,62	22,7	160,72
1004,71	659,09	90,31	852,41	173,07	135,26	141,79	24,7	169,29
973,97	743,49	130,95	1011,21	292,52	169,58	232,55	28,85	170,98
776,67	493,8	76,96	689,87	241,04	265,07	177,29	20,53	140,09
359,95	234,6	21,65	167,18	122,22	101	51,31	5,61	59,43
927,63	382,46	49,92	445,4	176,64	93,9	132,32	11,81	101,12
661,92	373,55	86,07	406,86	223,67	266,64	158,61	43,77	140,09
517,33	208,06	31,49	292,26	188,43	74,58	138,7	14,73	80,42
467,48	213,47	50,87	364,05	437,28	94,19	241,82	17,57	78,87
432,34	196,59	54,58	290,38	11,13	8,47	15,17	5,87	74
427,13	217,08	42,85	310,63	176,76	172,33	121,22	12,35	94,8
369,67	104,06	112,8	235,73	199,51	203,23	85,43	7,2	63,3
245,11	51,55	32,33	53,03	305,84	158,21	206,08	9,55	75
375,69	139,26	129,18	265,24	225,8	133,73	83,69	9,49	81,3
341,72	206,24	47,78	357,96	152,45	154,42	154,46	12,45	107,51
452,21	209,34	42,21	339,92	324,47	569,86	437,16	12,08	62,54
489,02	292,29	51,03	445,62	264,92	282,48	76,09	25,36	155,9
415,08	865,77	63,68	253,27	380,06	366,29	177,22	17,15	80,8
218,99	146,81	33,73	235,78	141,66	199,41	93,77	4,37	64,92
311,35	186,54	59,32	290,72	189,51	323,02	290,86	9,76	75,27
546,03	284,89	76,77	528,96	188,71	75,83	120,66	24,54	110,33
149,04	49,91	13,27	122,47	40,66	74,37	56,47	2,23	41,5
415,39	195,69	46,63	284,81	146,84	239,91	259,8	13,45	63,35
317,8	175,77	46,1	318,63	118,73	70,19	164,99	10,7	83,48
406,11	204,82	59,03	406,34	148,59	202,49	143,92	15,62	97,42

MIP-2	MIP-3a	MIP-3b	RANTES	SCYB16	TARC	TECK	TNF-a	SDF1a
60,06	24,19	126,82	22,1	66,86	28,04	3821,54	90,1	15417
76,97	27,57	94,85	16,86	393,78	51,73	4096,12	113,38	21862,76
127,3	25,08	194,47	67,44	512,32	121,47	646,43	131,59	2974,02
70,05	20	71,81	62,11	131,6	42,92	704,74	115,75	2714,24
321,02	55,98	118,03	184,45	128,93	44,32	1356,22	973,86	10971,96
98,67	68,16	88,07	138,37	240	53,98	8085,89	352,76	31248,99
290,74	50,71	137,55	103,43	48,87	40,77	3568,34	712,55	6459,39
140	25,27	140,27	116,8	25,62	93	11600	563	1628,67
42,49	10,02	56,9	15,6	340,53	19,53	31,13	37,33	815,63
178,28	97,24	153,64	59,83	271,49	66,31	1550,06	868,82	8801,58
78,47	14,28	96,23	12,6	486,84	45,94	257,87	80,97	26749,2
472,56	110,93	197,79	40,48	360,85	164,3	38878,72	340,54	2974,02
89,25	31,76	166,32	88,62	226,83	80,51	5688,07	101,22	2974,02
81,47	119,52	128,09	40,34	166,98	24,88	5299,44	142,07	6410,34
129,1	114,26	119,98	55,72	265,25	48,08	10549,62	294,23	16060,7
84,76	90,67	124,26	48,8	174,82	20,71	10426,2	216,59	22726,18
151,79	50,71	86,16	185,2	84,75	37,84	1193,85	299,5	9585,65
45,77	35,8	109,73	12,95	362,43	84,59	1728,24	50	8043,08
74,26	56,46	95,31	37,94	343,23	86,01	8309,52	118,11	24285,49
129,17	167,87	248,39	168,17	13,89	232,06	57486,49	304,68	2974,02
188,75	52,87	213,99	57,36	172,94	162,66	19003,91	330,66	2974,02
260,05	87,72	262,22	140,82	256,39	327,18	15259,57	439,56	2974,02
140,37	102,8	188,17	100,14	179,59	140,58	18553,5	212,25	2974,02
38,73	54,9	105,96	33,96	85,51	28,03	1359,65	29,94	2974,02
90,46	72,11	245,43	142,77	120,88	69,65	1009,88	1034,38	2974,02
319,75	102,69	194,47	37,56	60,73	41,75	4701,16	693,8	2974,02
54,36	27,66	123,03	60,44	354,28	72,75	5858,1	98,78	2974,02
206,16	73,33	132,95	130,81	109,86	61,91	11935,14	517,83	7297,41
115,7	53	129	58	8,04	56	5000	291	276,96
78,92	58,67	101,02	29,02	283,55	51,94	5159,54	171,44	47013,03
98,53	13,97	97,84	30,29	423,47	64,81	336,09	90,1	17013,1
110,79	10,52	88,07	56,63	164,37	17,6	704,74	76,44	1448,39
78,47	60,22	94,62	39,15	183,36	120,95	5925,68	99,34	16859,47
115,29	53,88	101,02	42,95	213,04	59,87	17834,97	484,26	13704,45
82,07	59,14	136,3	38,91	101,83	26,92	5586,5	196,37	16918,13
102,2	105,23	139,38	87,75	215,86	77,86	8127,39	134,02	53263,35
107,87	113,8	271,78	99,64	281,81	31,43	5316,96	84,04	53263,35
46,38	36,21	51,6	31,19	65,96	31,27	2241,15	40,57	2723,37
60,48	75,35	83,6	67,95	129,96	27,59	6406,31	144,99	174326,12
124,39	54,63	121,71	85,4	149,97	47,78	2325,68	172,85	19701,77
14,56	24,42	40,42	14,03	29,49	20,68	1929,11	5,77	776,91
62,6	70,79	121,71	42,47	104,53	37,75	6772,15	52,44	8000,61
58,92	53,93	73,53	65,83	154,54	36,84	4254,49	130,15	10856,69
82,01	41,77	111,67	56,67	102,56	41,64	1510,85	168,95	3092,12

	WT-ALS_asympt<br>P.adj	WT-ALS_sympt<br>P.adj	WT-ALS_term<br>P.adj	ALS_asympt-ALS_sympt<br>P.adj
**BCA-1**	0,9936103	0,2190347	0,2764427	0,2123265
**CTACK**	0,81946162	0,40864267	**0,01797035**	0,61985818
**ENA-78**	0,20538318	0,23496685	0,06441764	0,86496482
**Eotaxin**	0,483198235	0,260054378	**0,009651645**	0,548089799
**Eotaxin-2**	0,153518	0,1322052	0,1737598	0,9282127
**Fractalkine**	0,9329869	0,4707974	0,1142175	0,5786679
**GM-CSF**	0,5508749	0,9412737	0,6397124	0,4881819
**I-309**	0,6770352	0,7182319	0,348532	0,8804299
**IFN-g**	1	0,9328452	0,9164839	0,925824
**IL-1b**	0,11492406	0,17958979	0,06841873	0,72331175
**IL-2**	0,869673	1	0,9567104	0,5324947
**IL-4**	0,9945748	1	0,4380938	0,8948755
**IL-6**	0,9840268	0,28921	0,1472009	0,4036419
**IL-10**	0,83014855	0,77869706	0,05261011	0,8759006
**IL-16**	0,3756715	0,3795334	0,3545441	0,8089991
**IP-10**	0,8839176	0,4328523	0,3533916	0,6056626
**I-TAC**	0,4608373	0,9359955	0,3264501	0,4185257
**KC**	0,9971268	0,848733	0,9427003	1
**MCP-1**	0,9840268	0,9748076	0,2550826	0,8568967
**MCP-3**	0,30696562	**0,02616645**	0,42480004	0,51345577
**MCP-5**	0,9489171	1	1	1
**MDC**	0,5736539	0,4278652	0,2146184	0,9527218
**MIP-1a**	0,5547648	0,5745397	0,3152779	0,5863971
**MIP-1b**	0,4333515	0,4804319	0,3859814	0,7685001
**MIP-2**	0,781102	0,4862323	0,5126976	0,7374249
**MIP-3a**	0,3607885	0,610635	0,2962192	0,4166542
**MIP-3b**	0,8301114	0,3154751	0,5286517	0,384781
**RANTES**	0,9971874	0,9902113	1	0,8524126
**SCYB16**	0,6936323	1	0,525698	0,9904156
**TARC**	0,67380874	0,61281357	0,09819609	0,89012681
**TECK**	0,38694572	0,86099054	0,08869744	0,48453008
**TNF-a**	1	0,8558496	1	0,9057422
**SDF1a**	1	0,781456	0,9650919	1

ALS_asympt-ALS_term	ALS_sympt-ALS_term	WT		ALS_a
P.adj	P.adj	mean	sd	mean
0,3007378	0,8206245	13044,19	9074,852	15195,48
0,12306268	0,26965749	1393,076	962,7065	1448,498
0,74271846	0,67694618	7365,28	14194,058	13500,55
0,198120792	0,393114981	517,4776	255,4854	609,3783
0,8106882	0,8692938	4604,455	2968,288	7740,233
0,2681483	0,5770016	130,4829	46,42893	155,2033
0,4987545	0,6765544	16,8781	11,303866	11,21167
0,7436836	0,6620881	33,81714	27,08704	33,52
0,3235125	0,4719459	24,58286	10,19741	24,29833
0,73652255	0,81278189	257,1438	181,5584	791,3317
0,3728193	1	36,475	33,75868	19,115
0,7317339	0,5279494	5,544286	2,683706	7,558333
0,2775609	0,6611287	25,2581	15,01339	35,26667
0,30736199	0,22020014	237,1719	140,7767	279,885
0,807379	0,8412716	785,2152	550,8577	1230,065
0,514864	0,7638634	421,2852	162,9299	468,7467
0,2096843	0,4254502	235,1786	167,23742	161,98
1	0,9782973	54,82333	27,69157	56,81
0,362739	0,4045941	305,4876	111,5006	313,9867
0,53284856	0,23864017	198,28	101,76032	260,8583
1	0,9617626	184,0262	128,21443	188,1517
0,8035024	0,9485478	152,8452	95,61586	195,2
0,4871036	0,7043239	13,60286	9,071454	10,5
0,399695	0,2893017	85,30238	26,9439	69,48
0,7333674	0,8299712	97,55429	64,42019	125,67833
0,1160683	0,2713756	57,91524	27,66368	36,83
1	0,9698204	122,1481	56,01849	115,675
0,9895833	1	59,59143	33,47884	81,88833
1	1	159,219	105,5836	245,5817
0,57591373	0,41412549	48,81048	24,26026	57,07667
**0,04998575**	0,10628548	4966,454	4099,746	3118,49
0,9120327	0,8343114	224,641	255,6535	296,24
0,8665299	0,7086373	21830,062	38724,897	14198,162

sympt	ALS_sympt		ALS_term	
sd	mean	sd	mean	sd
16090,126	21337,46	9172,58	19368,71	8315,664
560,9902	2069,206	1425,4899	3952,98	2517,6657
11993,037	11889,84	12581,291	12565,28	9954,854
152,3668	702,23	326,001	923,1211	341,383
3929,441	7292,324	2550,458	6788,471	3000,087
81,74118	203,6213	132,32397	264,1	151,70748
5,620617	22,79375	21,893676	26,47444	16,743884
14,72939	26,6025	12,5766	40,72222	13,98379
14,44561	30,41875	26,85049	48,18556	32,10753
625,8567	333,2138	129,4604	352,3678	104,2566
12,51516	30,7175	18,42373	31,82	17,08277
6,207055	7,47125	5,922621	8,691111	4,474144
36,06917	78,74625	122,03994	45,51778	23,08121
139,8553	372,3662	364,501	475,6411	202,5495
385,4321	1189,6263	704,2283	1041,2778	439,9186
186,6536	540,3537	225,8793	650,6733	302,7032
82,00026	242,5662	175,05641	375,6422	237,65387
58,1978	118,27875	189,63116	61,41444	35,76607
123,8125	375,6125	230,3467	548,2922	297,7257
73,88999	305,8363	93,43355	245,9556	105,56895
133,82027	205,0412	98,82109	203,0444	100,86897
88,01125	200,72	55,54578	216,5167	112,41685
4,941838	16,68625	11,371195	18,88444	8,938771
20,74449	89,1725	66,6388	112,00667	49,33361
98,65417	171,6575	144,45066	133,78	64,29241
20,07546	57,46625	44,96069	84,35111	41,10884
43,49313	134,5987	43,01899	160,9122	68,01274
66,50164	59,7125	39,49058	89,67778	61,68774
174,1747	241,0012	157,9419	205,9211	113,8653
32,8358	66,905	47,0338	126,63444	100,94473
2869,424	8359,204	12888,234	15834,544	16881,192
345,8269	355,8125	320,9519	251,7311	117,1243
11128,635	7101,606	8395,14	10288,576	8685,277
